# Affective phenotypes in heterozygous *LRRK2* R1441G knock-in mice

**DOI:** 10.3389/fgene.2025.1629897

**Published:** 2025-08-29

**Authors:** Marcus H. F. Ng, Jimmy W. Y. Lam, Zoe Y. K. Choi, Hui-Fang Liu, Philip W. L. Ho, Benson W. M. Lau, Benjamin K. Yee

**Affiliations:** ^1^ Department of Rehabilitation Sciences, The Hong Kong Polytechnic University, Hung Hom, Hong Kong SAR, China; ^2^ Mental Health Research Centre, The Hong Kong Polytechnic University, Hung Hom, Hong Kong SAR, China

**Keywords:** animal model, Parkinson’s disease, leucine-rich repeat kinase 2, behavioural phenotyping, depression

## Abstract

Several missense mutations in the *LRRK2* gene are linked to familial Parkinson’s disease (PD). Although *LRRK2* mutant mouse models typically lack gross motor impairments, their contribution to non-motor PD symptoms remains largely underexplored. In this study, we showed that the R1441G missense mutation promoted behavioural despair in the forced swim test (FST) and led to anhedonia, reflected in reduced sucrose preference, while the typical expression of helplessness in avoidance learning, induced by undermining locus of control, was unaffected. Notably, these depressive phenotypes emerged predominantly in heterozygous R1441G knock-in (KI) mice, and a similar dominant negative phenotype was evident in the elevated plus maze, with heterozygous mutants exhibiting lower anxiety than wild-type (WT) mice. Together, these results suggest that the R1441G mutation may impact select dimensions of affective function in prodromal adult mice, irrespective of sex. In contrast, no overt behavioural phenotypes were detected in cognitive, social, or motor domains, including associative learning, hippocampus-dependent spatial learning, sensorimotor gating, social interaction, motor coordination, grip strength, or spontaneous locomotor activity. Further investigation is warranted to dissect the mechanisms underlying the domain-specific and seemingly dominant-negative behavioural effects of the R1441G mutation, especially in comparison to the behavioural phenotypes associated with other models of *LRRK2* mutations.

## 1 Introduction

The hallmark motor symptoms of Parkinson’s disease (PD), including bradykinesia, rigidity, and resting tremor, are primarily attributed to the degeneration of nigrostriatal dopaminergic neurons and the resulting depletion of striatal dopamine (DA) (Burke and O’Malley, 2013). Increasingly, the significance of non-motor dysfunctions is being recognised on both pathophysiological and clinical grounds (Li et al., 2024; Schapira et al., 2017; Tanner and Ostrem, 2024). As PD progresses, many individuals experience non-motor symptoms, including cognitive decline, depression, apathy, anxiety, sensory impairments, and autonomic dysfunction (Barone et al., 2009), all of which significantly undermine quality of life (Chaudhuri and Schapira, 2009). Notably, these non-motor symptoms often precede the onset of Parkinsonian motor signs (Schapira et al., 2017). Their early emergence highlights the potential for aiding early diagnosis and intervention and investigating the pathophysiological mechanisms during the prodromal phase of PD (Berg et al., 2021).

Among the genetic risk factors identified in familial PD, LRRK2 mutations represent the most common monogenic cause (Kluss et al., 2019; Lesage and Brice, 2009). The *LRRK2* gene encodes a large multidomain protein with kinase and GTPase activities, playing critical roles in neuronal processes such as synaptic vesicle trafficking, dopamine homeostasis, and cytoskeletal dynamics (Belluzzi et al., 2012; Berwick and Harvey, 2011). Interestingly, asymptomatic LRRK2 mutation carriers have been reported to exhibit increased DA turnover and subtle neuropsychiatric signs (Mirelman et al., 2015; Sossi et al., 2010), suggesting that LRRK2 mutations associated with PD may contribute to non-motor phenotypes. Non-motor symptoms such as mood disturbances and olfactory dysfunction have been observed in patients carrying LRRK2 mutations, suggesting that these mutations may be associated with a broader clinical phenotype (Deng et al., 2020; Chahine et al., 2025). However, it remains controversial whether the prevalence or severity of these non-motor symptoms is greater in LRRK2 mutation carriers than in non-carriers.

Among LRRK2 mutations, the G2019S variant in the kinase domain and missense mutations at the R1441 residue (such as R1441C and R1441G) in the GTPase domain are particularly prevalent (Simpson et al., 2022). These mutations are believed to enhance *LRRK2* kinase activity *via* distinct, complementary mechanisms. While the G2019S mutation increases catalytic activity within the kinase domain, R1441 mutations potentiate kinase activation by impairing GTPase function (Fan et al., 2021; Henry et al., 2015). Notably, Fan et al. (2021) further proposed that this gain of function in kinase activity is more pronounced with R1441G mutations than with G2019S. Nevertheless, the specific link between LRRK2 mutations and non-motor symptoms in PD remains unclear.

Mutant models of LRRK2 mutations have been pivotal in exploring their causative roles in PD pathogenesis. Although several mutant LRRK2 mouse lines have been generated, most studies have focused on motor and neuropathological features (Chen et al., 2012; Domenicale et al., 2022; Longo et al., 2017; Mercatelli et al., 2025; Xenias et al., 2022). Few studies have systematically examined non-motor behavioural phenotypes, and there is a bias toward investigating the G2019S mutation, with mixed results reported (see Table 1 in the Discussion section). By comparison, there is a distinct paucity of behavioural data for R1441C and R1441G mutants.

**TABLE 1:** Non-motor behavioural studies in LRRK2 mutation rodent models.

			Age range (months)	Depression-related						Anxiety-related					Learnt fear		Memory					Attention		Motivation			Others		
				Forced swim test	Tail suspension test	Social defeat response	Learnt helplessness	Sucrose preference test	Sucrose splash test	Elevated plus maze	Marble burying task	Light dark exploration	OF - centre exploration	NSF test	Conditioned freezing	Passive avoidance	Y-maze	Spontaneous alternation	Water maze	Object recognition	Discrimination reversal	Prepulse inhibition	5-CSRTT	Operant conditioning	Devaluation	Progressive ratio	Olfaction	Formalin test	Social interaction
R1441G	Tg	Bichler et al. (2013)	4∼21	=	=					=			=			=											=	=	
		Shaikh et al. (2015) ^a^	3∼12																=			=							
	KI	Ho et al. (2023)	3 and 14								↓																		
		Present study^b^	4∼8	↑			=	↑		↓			=		=		=		=			=							=
R1441C	Tg	Sloan et al. (2016) ^a^	3∼6															=											
			18∼21															↓											
	KI	Giesert et al. (2017)	2∼3	↓	↓								=							=									
			24∼26																								↓		
G2019S	Tg	Herzig et al. (2012)	2∼4							=		=	=						=										
		Sloan et al. (2016) ^a^	3∼6															=											
			18∼21															↓											
		Adeosun et al. (2017)	9∼10														↓	=	↓										
		Lim et al. (2018)	2∼5	↑	=			=		=		=																	
			10∼12	↑	↑			↑		↑		↑																	
			15∼19					↑		↑		↑																	
		Skiteva et al. (2022)	10∼12							=		=	=																
		Kim et al. (2022)	1.5∼2.5	↑	↑							=	=																
	KI	Yue et al. (2015) ^b^	6 and 12							=		=	=		=				=	=									
		Matikainen-Ankney et al. (2018)	2∼3			↓		=	↓	=																			
		Guevara et al. (2020)	2∼3			↑		↓																					
		Hussein et al. (2022)	2∼3																		=		↓	↓	↓	=			
		Guevara et al. (2024)	2										=	↑															=

Studies of non-motor behavioural phenotypes in LRRK2 mutation rodent models are summarised and organised by mutation type (R1441G, R1441C, and G2019S) and mutation model [Tg (transgenic); KI (knock-in)]. To facilitate comparison, the age of tested mice was converted into unit of “months”. Behavioural tests are classified into seven domains: depression, anxiety, learnt fear, memory, attention, motivation, and other non-motor behaviours. Blank cells indicate no data reported. For affective domains (i.e., depressive and anxiety-related behaviours), “↑” denotes more depressive/anxiety-like behaviours, while “↓” indicates the anti-depressive or anxiolytic-like phenotypes. “↓” in the memory; attention and motivation domains represent loss of function. “=” in all cells indicates no significant difference compared to their respective controls. Abbreviations: NSF, novelty suppressed feeding; 5-CSRTT, five-choice serial-reaction time task.

^a^ Indicates that the model was generated in rats instead of mice.

^b^ Indicates studies with comparing homozygous, heterozygous, and wild-type genotypes.

Early mouse models of the R1441G mutation included transgenic lines with *LRRK2* ^R1441G^ overexpressed at levels 5–10 times higher than endogenous wild-type (WT) LRRK2 (Li et al., 2009). However, these mutant mice did not exhibit significant alterations in non-motor behaviours, such as depression- and anxiety-like responses or impairments in learning and memory (Bichler et al., 2013). A similar transgenic rat model likewise failed to show deficits in sensorimotor gating or spatial memory (Shaikh et al., 2015). To better model the genetic context of human R1441G carriers, Liu et al. (2014) generated knock-in (KI) mice expressing *LRRK2* ^R1441G^ under endogenous regulatory control. Although homozygous KI mice (LRRK2^R1441G/R1441G^) exhibited impaired synaptic dopamine uptake and disrupted vesicle trafficking (changes that may contribute to dopaminergic dysfunction), no baseline behavioural abnormalities were observed. Only under pharmacological challenge did these mutants exhibit increased sensitivity to reserpine-induced locomotor suppression compared with wild-type controls (Liu et al., 2014). A spontaneous behavioural phenotype was later reported by the same authors in the form of a pronounced deficit in marble burying behaviour (Ho et al., 2023; see their Figure 5; Supplementary Figure S2). However, it remains uncertain whether this phenotype reflected reduced novelty-induced anxiety (neophobia) or an underlying motivational impairment (Njung’e and Handley, 1991; Thomas et al., 2009).

In addition to the limited behavioural scope employed, Ho, Liu and their colleagues (e.g., Liu et al., 2014; Liu et al., 2017; Ho et al., 2022; Ho et al., 2023) also chose to focus exclusively on homozygous KI mice, thus leaving a gap in data concerning heterozygous carriers of the R1441G mutation. This omission is particularly notable given that the heterozygous genotype (*LRRK2* ^R1441G/WT^) arguably better approximates the majority of PD patients carrying this mutation (Gorostidi et al., 2009; Vinagre-Aragón et al., 2021). The importance of incorporating heterozygous models is underscored by evidence that the LRRK2 G2019S heterozygous knock-in genotype (LRRK2^G2019S/WT^) exhibits distinct phenotypes compared with its homozygous counterpart (Yue et al., 2015), including differences in amphetamine-induced dopamine release (see their Figure 3e), mitochondrial oxidative phosphorylation (see their Figure 15b1), and PINK1 expression (see their Figure 15b2).

The present study offers the first comprehensive behavioural assays of the *LRRK2* R1441G mutation KI mouse model generated by Ho and others, directly comparing homozygous (denoted as KI^+/+^) and heterozygous (KI^+/−^) genotypes against WT littermates. Both male and female mice were included to evaluate the potential contribution of the R1441G mutation to sex-dependent clinical features of PD. Previous research suggests that affective symptoms are more prevalent among women, whereas men more often exhibit pronounced cognitive deficits (Maas et al., 2024). Animals were tested across a battery of behavioural assays targeting anxiety, depression (behavioural despair, anhedonia, and learnt helplessness (LH)), sensory gating, spatial learning and memory, fear conditioning, and social interaction. Motor coordination and muscle strength were also assessed to evaluate the potential suitability of these mutant lines as prodromal models of PD during adulthood (4–6 months of age).

## 2 Methods

### 2.1 Subjects

The *LRRK2* ^R1441G^ KI mouse line was originally generated by Liu et al. (2014), in which the R1441G mutation was introduced to the ROC GTPase domain of LRRK2 (c. 4321 C > G). The mouse line was maintained at the University of Hong Kong. Mice in the present study were obtained by intercrossing heterozygous mutants (KI^+/−^) to obtain three genotypes: homozygous (KI^+/+^), heterozygous (KI^+/−^), and wildtype (WT), as littermates in a 1:2:1 ratio.

Seventy-two adult offspring derived from over ten independent litters were transported to The Hong Kong Polytechnic University for the planned experiments. They were shipped in two independent cohorts (A and B), balanced with respect to genotypes and sex (n = 6 per genotype per sex per cohort). All mice were acclimatised to the Central Animal Facilities at The Hong Kong Polytechnic University for at least 2 weeks prior to experimentation.

The mice were housed in Makrolon® cages (Tecniplast, Italy) in a climate-controlled vivarium (22 °C ± 1 °C and 55% ± 5% RH), maintained on a 12/12 h light–dark cycle, with lights off from 20:00 to 07:59 h. Food and water were available *ad libitum*, unless otherwise specified. The experimenters were licensed by the Department of Health to perform animal experiments in accordance with the Animals (Control of Experiments) Ordinance (Cap. 340) under the Laws of Hong Kong. Institutional approval was granted by the University’s Animal Subjects Ethics Committee (PolyU ASESC: 23-24/731-RS-R-OTHERS). Every effort was made to comply with the European Union Directive 2010/63/EU on animal welfare in scientific research and the *Guide for the Care and Use of Laboratory Animals* (U.S. National Academies, 2011).

### 2.2 Genotyping

**FIGURE 1:**
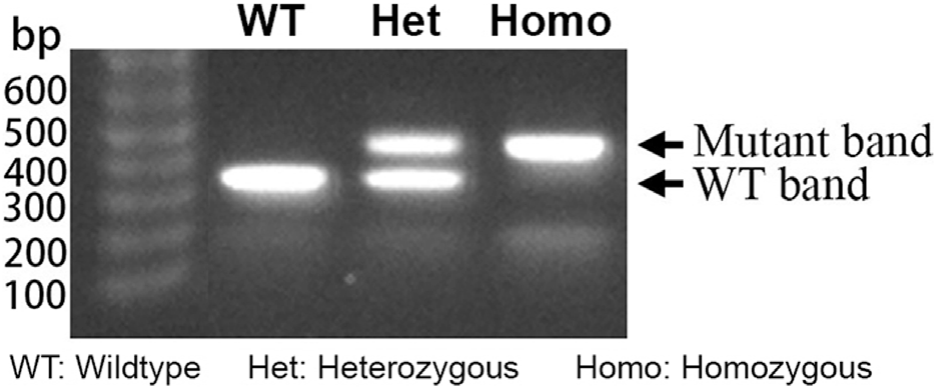
Typical gel electrophoresis of PCR products from DNA extracted from KI^+/−^, KI^+/+^, and WT mice. The heterozygous KI (KI^+/−^) lane shows bands at 462 bp and 380 bp, whereas the homozygous KI (KI^+/+^) and wildtype (WT) each display a single amplicon at 462 bp and 380 bp, respectively, consistent with expected fragment sizes for each genotype.

Genomic DNA was extracted from ear punches. Genotyping was performed using the KAPA Express Extract Kit (KR0383, Kapa Biosystems) as previously described in full (Liu et al., 2014). The resulting supernatant served as the template for genotyping through PCR using the SapphireAmp^®^ Fast PCR Master Mix (RR350A, TaKaRa) (Figure 1). The primer forward (*F*) and reverse (*R*) sequences for mouse genotyping were, respectively, *F*: 5′-ACG​CTG​CTG​TGT​CAC​ACG​GTT-3′ and R: 5′-TCC​GAA​GCT​TTG​CCA​GCG​CAT-3′.

### 2.3 Behavioural procedures

**FIGURE 2:**
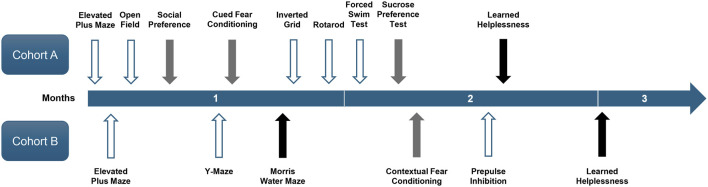
Timeline of behavioural tests. The timeline shows the chronological sequence of behavioural tests conducted on two cohorts of mice over the 2.5-month period. Arrows indicate the start of each test, with their colour denoting test duration: white = 2 days, grey = approximately 1 week, and black = approximately 2 weeks.

Behavioural testing began when the mice were approximately 4–6 months old. It was completed in 2.5 months and included 13 tests. The two cohorts of mice underwent different sequences of tests as depicted in Figure 2. The test sequence was generally arranged according to the degrees of stress involved, with the most severe test (learnt helplessness) placed at the end. Although no aversive stimuli were delivered, the two motor tests (inverted grid and rotarod) were scheduled in the middle of the test sequence for Cohort A. This was intended to generate more representative validation for the absence of motor impairment that might confound other assays. The sucrose preference test (SPT) was conducted after the forced swim test (FST) in order to delay the switch to single housing (see Section 2.3.10). Only the elevated plus maze test and learnt helplessness in the conditioned active avoidance paradigm were completed with animals of both cohorts. Otherwise, cohorts A and B completed distinct sets of tests. To minimise interference from olfactory cues from the opposite sex, male and female mice were always evaluated on separate days. Hence, all animals were left undisturbed for at least 24 h between tests. All tests were conducted between 09:00 and 19:00 h during the light phase under dim lighting in the testing rooms. To minimise influence of acute transport stress, animals were always acclimatised to the testing room for at least 30 min before testing.

#### 2.3.1 Elevated plus maze

The elevated plus maze (EPM) was constructed from grey acrylic and elevated 65 cm above the floor. It comprised four arms, each measuring 30 cm in length, connected to a 5 cm × 5 cm central platform. Two opposing arms were surrounded by 30-cm-high walls, while the other two arms were bordered only by a narrow 5 mm rim. To begin the test, the mouse was placed in the centre with its head facing an open arm and then allowed to explore freely for 5 min. A video camera (FDR-X3000, Sony, Japan) positioned directly above the maze recorded the session for subsequent analysis using EthoVision XT (v11.5, Noldus, Netherlands), which tracked the animals’ movements and time spent inside each arm. Anxiety was indexed by the proportion of time in the open arms relative to all four arms.

#### 2.3.2 Open-field test of locomotor activity

The open-field test was performed using four 40 × 40 cm grey acrylic arenas with 33 cm perimeter walls. Mice were initially placed in the centre of the open-field arena and allowed to explore freely for 30 min. The mouse’s movement within each arena was tracked using EthoVision XT from video recordings. Distance travelled was recorded in successive 5-min bins to measure levels of locomotor activity and habituation. The average minimum distance from the arena walls was also computed to index thigmotaxis, which may reflect anxiety (Simon et al., 1994). This metric has the advantage of avoiding the arbitrary designation of a central zone.

#### 2.3.3 Social preference test

The apparatus was a 60 cm × 40 cm opaque Plexiglas box divided by transparent walls into three 20 cm × 40 cm chambers. Each partition had a 9 cm × 6 cm opening connecting the side chambers to the central chamber. Each side chamber contained an upside-down cylindrical metal mesh container (9.8 cm high, 9 cm diameter, 5 mm mesh). A weight was placed on top of each container to prevent it from tipping over and the mouse from climbing onto it. The test comprised three 10-min phases: habituation, social interaction, and social novelty. During habituation, each mouse explored the entire box surface freely for 10 min without the metal mesh container. For the social interaction phase, the mouse was briefly removed, then returned after placing an unfamiliar, same-sex, age-matched C57BL/6J mouse and a plastic toy mouse in the mesh containers in the side chambers. In the social novelty phase, the toy was replaced with another unfamiliar, same-sex, age-matched mouse. The placement of the mouse and the toy (or unfamiliar mouse) was counterbalanced across subjects. EthoVision XT was used to track time spent in each chamber. A preference ratio contrasting the time spent in the two side chambers was calculated as (mouse)/(mouse + toy) for social interaction and (unfamiliar)/(unfamiliar + familiar mouse) for social novelty to index relative preferences. A ratio exceeding 50% indicates a preference for the mouse (over the toy) or the novel mouse (over the familiar mouse) and social novelty in the social interaction test and social novelty test, respectively.

#### 2.3.4 Y-maze test of spatial familiarity

The Y-maze, made of grey acrylic, consisted of three 5-cm-wide × 30-cm-long arms connected at a central equilateral triangular area. The maze was surrounded by a 5-cm-high perimeter wall and equipped with retractable panels, positioned 5 cm into each arm, to control access. The arm nearest to the testing room door was designated as the “Start” arm. The other two arms served as familiar (F) and novel (N) arms, designated in a counterbalanced manner across mice. To begin, the mouse was placed in the Start arm and allowed to explore the maze for 6 min, with access to only arm F (access to arm N was blocked). Afterwards, the mouse was placed in an opaque waiting cage in an adjacent room for 30 min (delay period). Next, it was reintroduced to the maze with free access to both F and N arms and observed for 3 min. EthoVision XT was used to track mouse locations in both phases. Novel arm preference in the test phase was indexed by (N−F)/(N + F) based on time spent in each arm, with values above 0 indicating a preference for the novel over the familiar arms.

#### 2.3.5 Cued fear conditioning

Two conditioned freezing chambers (MED-VFC-USB-M, Med Associates, VT, United States) were used. Descriptions of their construction and dimensions are available at https://med-associates.com/product/nir-video-fear-conditioning-vfc-system-for-general-use. In brief, each chamber was equipped with a grid floor made from stainless-steel rods (4 mm in diameter, spaced 10 mm apart centre-to-centre), through which scrambled electric shocks can be administered. The conditioned stimulus (CS) was an 86-dB, 30-s pure tone at 5 kHz delivered from a speaker mounted on the chamber wall. Background lighting in the chamber was provided by an incandescent light bulb (i.e., a house light in the visible wavelength) and an infrared light source mounted on the wall. All sessions were recorded using an infrared camera. Freezing was estimated based on immobility time detected using Med Associates image analysis software, applying the recommended settings for mice.

##### 2.3.5.1 Conditioning (day 1)

The mice received three discrete trials of CS–US (unconditioned stimulus) pairings after entering the chambers. Each trial began with the 30-s tone CS, followed immediately by a 1-s, 0.25 mA foot shock US. Each CS–US pairing was preceded and followed by a 180-s inter-trial interval (ITI).

##### 2.3.5.2 Context-freezing test (day 2)

The mice were returned to the conditioning chambers and left undisturbed for 8 min to gauge the freezing response to the contextual background against which the CS–US pairings took place 24 h before.

##### 2.3.5.3 CS tests (days 3–5)

To better isolate the conditioned freezing response specific to the CS, the chamber’s context was modified. A distinct odour was introduced by placing 10 g of commercial freeze-dried coffee powder (UCC The Blend 117 Instant Coffee, Ueshima Coffee Co., Ltd., Japan) inside the cubicle under the ventilation fan 30 min before the start of the CS test. A curved opaque Plexiglas backwall was added to the back of the chamber, and the metal grid was covered with a Plexiglas floor. Finally, the house light was kept off throughout the tests. Two minutes after the mice were placed inside the modified chamber, the CS tone was activated continuously for 8 min. No shock was delivered. Percentage of time spent freezing was computed for each 1-min bin. The conditioned fear response to the CS was indexed by the elevation of immobility time observed after CS onset.

#### 2.3.6 Morris water maze of reference memory

A galvanised steel circular tank (170 cm diameter, 36 cm high) was positioned in the middle of a well-lit testing room, enriched with distal visual cues. It was refilled to 31 cm with water at 23 °C ± 1 °C. A transparent Plexiglas cylinder (12 cm in diameter, 30 cm high) with a roughened top surface to facilitate climbing served as the escape platform. Four equidistant points along the tank’s circumference (N, E, S, and W) served as the variable start positions. At the start of each trial, the mouse was lowered into the water at one of these start points with its head facing the tank wall. The tank was conceptually divided into four equal quadrants (NE, SE, SW, and NW) by two imaginary orthogonal lines intersecting at the centre. A ceiling-mounted camera recorded all trials for swim path tracking and analysis using EthoVision XT.

To acclimate the mice to the maze and reduce swimming-induced stress, pre-training was conducted prior to formal reference memory training. Pre-training consisted of three consecutive trials in which the escape platform was made visible by a vertical pole mounted at its centre, extending above the water surface. Throughout these trials, the platform remained fixed at the centre of the maze. To begin a trial, the mouse was gently placed in water at one of four start positions (N, E, S, and W) and given 60 s to find the escape platform. If a mouse failed to escape onto the platform within the allotted time, it was gently guided to the platform by the experimenter. The mouse remained on the platform for 15 s before the next trial. The ITIs were approximately 15–20 s. All mice learnt to swim and climb onto the platform and showed improvement across the three pre-training trials.

Spatial reference memory was evaluated over the next 10 days. Mice were given two trials per day with the platform hidden underneath the water fixed in the centre (42.5 cm off the maze centre) of one of the four possible quadrants (NE, NW, SE, or SW) randomly assigned to individual mice. For every set of four consecutive trials, each mouse was randomly assigned a sequence of start positions (N, E, S, and W), with each position used exactly once. As described above, if a mouse failed to locate the platform within 60 s, it was gently guided to it by the experimenter. In all cases, the mouse remained on the platform for 15 s before being removed and dried for the next trial. The ITIs were approximately 15–20 s. Performance was tracked by time (latency) and distance travelled to reach the platform. A latency of 60 s was assigned for failed trials.

On day 11, memory retention was assessed using a probe test. The platform was removed, and the mice were allowed to swim freely for 60 s. Search patterns were quantified as the percentage of time and path length in each quadrant across successive 15-s bin. Search accuracy was further quantified by computing the average straight-line distance to the centre of the learnt escape platform location within each 15-s time bin.

#### 2.3.7 Inverted grid

A standard wire cage top was used, with its edges masked off to prevent the mouse from climbing over to the other side. To begin, the mouse was placed horizontally on top of the cage lid, which was then slowly rotated 180° until the mouse was hanging upside down, 40 cm above a surface cushioned with sawdust bedding. Each mouse was given three attempts, spaced 1 hour apart. For each attempt, the latency to fall was recorded, up to a maximum of 300 s (i.e., the mouse did not fall).

#### 2.3.8 Accelerating rotarod

Motor coordination was assessed using a rotarod apparatus (Model LE8205, Panlab, Harvard Apparatus). Up to five mice were simultaneously placed on the rotating drum, initially set at 4 rpm. Once all the mice stabilised, the rotation speed was linearly increased from 4 rpm to 40 rpm over a 5-min period. A test trial ended when a mouse fell or after 300 s had elapsed. Each mouse underwent three trials spaced 1 hour apart. Performance was indexed by the latency to fall.

#### 2.3.9 Porsolt forced swim test

The apparatus consisted of four glass cylinders (15 cm in diameter, 24 cm tall), each filled with water at 22 °C to a depth of 15 cm. The cylinders were aligned in a row for simultaneous video recording and were separated by cardboard partitions to prevent visual contact between mice. To test the hypothesis that the mutants exhibit a depression-like phenotype, we employed a 2-day FST paradigm, with a 10-min pre-test followed by a 10-min test 24 h later, as originally specified in the rat protocol by Porsolt et al. (1977). The 2-day FST better models depressive-like behaviour by enhancing immobility detection as a depression proxy in mutants (Porsolt et al., 1978a; 1978b; Cryan and Mombereau, 2004). Mice were gently placed in the water, and sessions were recorded using a clear side-view camera. EthoVision XT measured immobility time in 1-min bins, with automated scoring validated against manual ratings by two blinded observers using two randomly selected videos to ensure reliability. Immobility time (i.e., floating, the absence of struggling, and swimming) was quantified as a measure of behavioural despair and motivational deficit (unwillingness to maintain effortful escape).

#### 2.3.10 Sucrose preference test

Two days prior to testing, mice were switched to single housing and acclimated to drinking from two measuring tubes modified from 15-mL polypropylene Falcon^®^ tubes (Thermo Fisher). Each tube was ground through at the conical end to create a 2.5-mm-diameter opening. The tubes were securely placed on the cage lid, with their openings positioned 4 cm apart and 5 cm above the bedding to facilitate easy access and switching between the two tubes during drinking. Following habituation to single housing and use of the modified drinking tubes, a 2-day baseline period was initiated. Body weight and fluid consumption from two water-filled tubes were monitored daily at 16:00 h to ascertain that all mice drank (∼5 mL over the last 24 h) and exhibited no persistent side bias.

Sucrose preference was assessed over the next 4 days with one bottle containing a sucrose solution and the other containing filtered water. The relative placements of the two drinking tubes were initially counterbalanced between mice and then swapped on the next day. The concentration of the sucrose solution was reduced from 1.0% to 0.25% over 2-day intervals. Daily measurement of fluid consumption was conducted at 16:00 h when the tubes were weighed and refilled. Sucrose consumption expressed as a percentage of total fluid intake [sucrose/(sucrose + water)] × 100% daily was computed as a proxy for sucrose preference. A value >50% indicates a preference for the sucrose solution.

#### 2.3.11 Foreground contextual fear conditioning

The test was conducted in the same two Med Associates conditioned freezing chambers described in Section 2.3.5. In this paradigm, no discrete cues contingent on the shock unconditioned stimulus were present to compete for associative learning during fear conditioning. Consequently, contextual features (represented by the diffuse elements of the chamber) were expected to become solely associated with the US. Conditioned fear specific to the shock-paired context (Context A) was demonstrated by elevated freezing during re-exposure to Context A, relative to a neutral Context B.

##### 2.3.11.1 Conditioning (day 1)

Mice received four unsignalled foot shocks (1-s, 0.25 mA) in Context A, each preceded and followed by a 3-min shock-free interval.

##### 2.3.11.2 Differential freezing to shocked and neutral contexts (days 2–5)

On Day 2, mice were returned to the shock-paired context (Context A) for 4 min in the absence of any discrete stimuli. On Day 3, the mice were placed in a neutral context (Context B), modified as described for Day 3 of the cued fear conditioning test in Section 2.3.5. This Context A → Context B test sequence was repeated across Days 4 and 5.

#### 2.3.12 Prepulse inhibition of the acoustic startle reflex

The test was conducted using an SR-LAB Startle Response System, equipped with two mouse test chambers (San Diego Instruments, United States). Each sound-attenuated chamber contained a non-restrictive cylindrical acrylic enclosure (3.8 cm inner diameter, 8.9 cm length) permanently affixed horizontally to a lightweight acrylic platform. This housing assembly for the mouse rested atop a heavy base block. A piezoelectric sensor mounted underneath the enclosure detected startle responses by converting the mouse’s whole-body movement forces into digital electrical signals, sampled at 1-m intervals. A high-frequency loudspeaker positioned directly above the enclosure provided a 65-dB(A) background noise and delivered white noise acoustic stimuli with 0.2–1.0 m rise/fall times at defined intensities and durations. A ceiling-mounted house light ensured stable ambient illumination throughout the test session.

The test began with 2 min of acclimatisation, followed by 108 trials with variable ITIs (mean 15 ± 5 s). Each trial consisted of a 20-ms prepulse, an 80-ms inter-stimulus interval (ISI), and a 40-m pulse. Prepulse intensities were 65, 71, 77, or 83 dB (i.e., at +0, +6, +12, or +18 dB above 65-dB background, respectively), and pulse intensities were 100, 110, or 120 dB, yielding twelve prepulse–pulse combinations. Prepulse inhibition (PPI), defined as the attenuation of the startle response with increasing prepulse intensity, was calculated as a percentage reduction relative to the baseline startle response (i.e., prepulse set at +0 dB). Whole-body acceleration was measured *via* piezoelectric outputs (mV) over two 65-m windows following prepulse and pulse onset. %PPI was computed for the middle 96 trials, using prepulse intensities (+6, +12, and +18 dB) and pulse intensities (100, 110, and 120 dB). The first and final six trials (prepulse at +0 dB) were used to stabilise and assess startle habituation.

#### 2.3.13 Learnt helplessness in the active avoidance paradigm

The test was performed using two active-avoidance shuttle boxes (MED-APA-D1M, Med-Associates, VT, United States), each housed inside a separate sound-attenuating chest. System specifications are available at https://med-associates.com/product/shuttle-box-test-package. Each shuttle box consisted of two compartments (20.3 × 15.9 cm) interconnected *via* a central doorway in the shared wall. Access between compartments (i.e., shuttling) can be regulated by a guillotine door. Ambient illumination within the chamber was provided by a house light mounted on the rear wall of the cubicle. The stainless-steel grid floor, connected to a scrambler module, delivered 0.38 mA electric shocks as the unconditioned stimulus. A 30 s, 2.9 kHz, 70 dB tone from a Sonalert module served as the CS. Shuttles between compartments are recorded using an array of photobeams aligned along the front wall of the shuttle.

In this study, we modified Seligman and Maier’s (1967) triadic design by comparing only controllable (escapable) and uncontrollable (yoked, inescapable) electric foot shock pre-exposure conditions. Mice from cohorts A and B were randomly assigned to escapable or inescapable pre-exposure conditions (n = 6 per condition, per sex, per genotype). Allocation to pre-exposure conditions was also balanced across cohorts to avoid potential bias arising from their differing prior exposure to electric shocks in the cued (Cohort A) or foreground contextual fear conditioning (Cohort B) experiments.

On the day of shock pre-exposure, mice assigned to the escapable and inescapable conditions were run simultaneously in yoked pairs, counterbalanced across the two shuttle boxes to control for chamber-specific effects. In each pre-exposure session, one escapable mouse (able to terminate shocks by shuttling) was yoked to an inescapable partner who received identical shocks without control over their offset. Subsequent learning deficits in active avoidance learning (conducted in the same shuttle boxes), observed in the inescapable yoked group relative to the escapable group, reflect the development of learnt helplessness. This deficit was compared across genotypes to assess the impact of prior loss of control over aversive events.

##### 2.3.13.1 Habituation (day 1)

All mice were familiarised with their designated shuttle box in a 15-min session. The intercompartment door was programmed to close for 2 s with a 25% probability whenever a mouse shuttled spontaneously and reached the far end of the opposite compartment. At all other times, the door remained open.

##### 2.3.13.2 Shock pre-exposure (day 2)

Mice in the escapable condition underwent 30 trials of shock exposure with a mean ITI of 45 ± 35 s. Each session began with the placement of the mouse in the right compartment with the door closed. After the initial ITI, a 0.38 mA shock was administered. Two seconds later, the door opened. The shock continued until a shuttle was registered or until an additional 28 s had elapsed, at which point the door also closed. The next trial began after the ensuing ITI had elapsed. The yoked inescapable mouse, tested concurrently, received identical shocks; however, the door remained open throughout the session, permitting free shuttling with no programmed consequences.

##### 2.3.13.3 Conditioned active avoidance (days 3–4)

Mice from both pre-exposure conditions underwent identical training during this phase. Each daily session comprised 50 discrete trials, with a mean ITI of 45 ± 35 s, and the intercompartment door remained open throughout. Trials commenced with the onset of the Sonalert tone (CS). The foot shock (US) was programmed to begin 5 s after CS onset if no shuttle was registered. The maximum duration of the US was limited to 2 s. A shuttle response within the initial 5 s terminated the CS without any shock delivery and ended the trial, which was recorded as a successful avoidance. A shuttle during the co-presentation of CS and US (max. 2 s) was recorded as an escape, terminating both stimuli. Failure to shuttle before the US reached 2 s was recorded as an escape failure. Performance was quantified by shuttle latency (max 7 s), which served as a continuous metric capturing behavioural dynamics across all response types. Latency was averaged over successive blocks of 10 trials across the two test days.

### 2.4 Statistical analysis

Statistical analysis was performed using IBM SPSS Statistics for Windows (Version 26). All data were subjected to analysis of variance (ANOVA), tailored to the nature of the dependent variable derived from each individual test. Preliminary models included the between-subject factors: genotype (KI^+/−^, KI^+/+^, and WT) and sex (male and female), and within-subject factors such as time bins, trials, concentrations, or other repeated-measures factors specific to each test. To streamline statistical reporting, sex was excluded from final models if it did not interact significantly with genotype or contribute to higher-order interactions. Statistical significance was set at *p* = 0.05, and effect sizes were reported as partial eta squared (*η*_*p*_^2^). Fisher’s LSD *post hoc* pairwise comparisons were performed to aid interpretation of significant main effects and interaction terms identified in the overall ANOVA. Data are presented as the mean ± standard error (SE), extracted from the SPSS output. *Ad hoc* correlational analyses were also conducted to explore relationships between behavioural metrics across tests.

## 3 Results

### 3.1 Depression-like phenotypes in *LRRK2* ^R1441G^ KI mice

**FIGURE 3:**
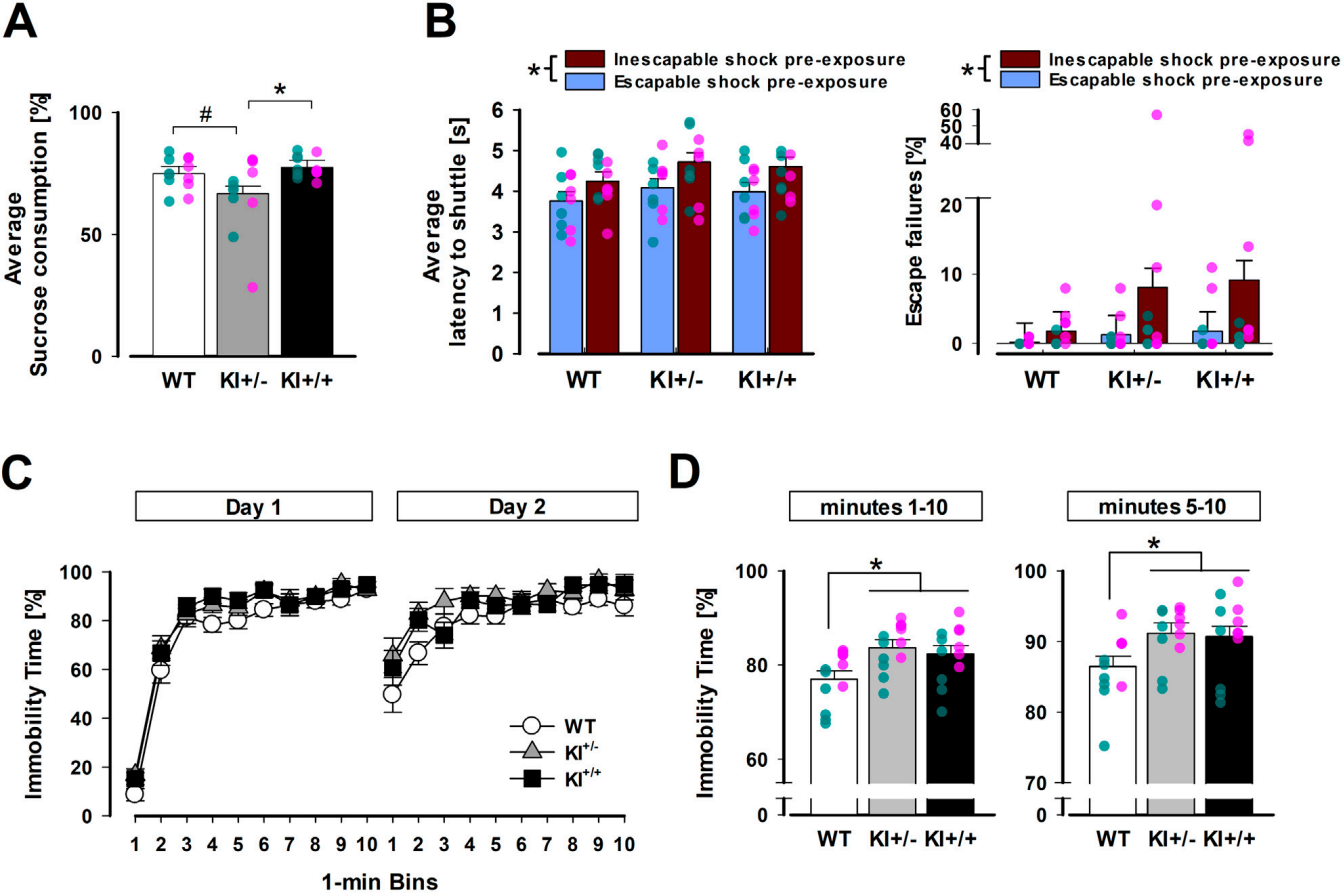
Evidence for depression-related behavioural phenotypes in heterozygous KI^+/−^ mice. **(A)** All mice showed a preference for the sucrose solution with the percentage of sucrose solution exceeding 50%, but a reduction was evident in the KI^+/−^ group, whose preference was significantly lower than KI^+/+^ mice (*p* < 0.05 *) and marginally below WT mice (*p* = 0.06 ^#^) based on pairwise comparisons by Fisher’s LSD. **(B)** Latency to shuttle (left) and percentage of escape failures (right) in the conditioned active avoidance task illustrate the impact of prior exposure to inescapable shock *versus* escapable shock. This difference resulted in a significant main effect of pre-exposure experience (*p < 0.01). **(C)** Immobility time (as a percentage) across successive 1-min bins on days 1 and 2 are separately depicted, showing earlier and stronger immobility in KI+/− and KI+/+ mice than WT mice (especially on Day 2). **(D)** Mean immobility time (as a percentage) in the FST is shown for the full 10-min test (bins 1–10, left) and the last 6 min (bins 5–10, right). Both KI+/− and KI+/+ mice exhibited significantly higher immobility than WT mice in *post hoc* pairwise comparisons (*p* < 0.05 *). The SPT and FST were conducted in mice from Cohort A. n = 12 (6♀+ 6♂) per genotype. The LH experiment included mice in both cohorts A and B. n = 12 (6♀+ 6♂) per genotype per pre-exposure condition. All data are presented as the mean ± SE. Overlaid scatter points represent data from individual mice (green dots = ♂; pink dots = ♀).

Depression-like phenotypes in *LRRK2* ^R1441G^ KI mice were detected in the SPT and FST. The anhedonia phenotype in the SPT was solely observed in the heterozygous KI^+/−^ mutants (Figure 3A). In contrast, the expression of behavioural despair in the FST was apparent in both KI^+/−^ and homozygous KI^+/+^ mutants. Nonetheless, the FST phenotype was numerically more prominent in KI^+/−^ mutants (Figure 3C). Neither mutant genotype, however, had significantly modified the expression of LH measured in active avoidance learning (Figure 3B).

#### 3.1.1 SPT

The presence of an anhedonia phenotype was supported by a 3 × 2 × 2 (genotype × sex × concentrations) ANOVA of percentage sucrose consumed, which yielded a significant main effect of genotype [*F*_(2,30)_ = 3.62, *p* < 0.05, *η*_*p*_^2^ = 0.19]. *Post hoc* Fisher’s LSD indicated it stemmed primarily from a lower sucrose preference in the KI^+/−^ mice relative to the KI^+/+^ mice [*p* < 0.05] and WT mice [*p* = 0.06]. Although the latter contrast failed to reach statistical significance, it was clear that KI^+/+^ and WT mice exhibited highly similar levels of sucrose preference. As expected, sucrose preference fell with decreasing sucrose concentration [*F*_(1,30)_ = 19.36, *p* < 0.001, *η*_*p*_^2^ = 0.39], but the genotype effect did not significantly depend on it [genotype × concentrations: *F*_(2,30)_ = 1.74, *p* = 0.19, *η*_*p*_^2^ = 0.10]. Instead, evidence indicated that the influence of sucrose concentration on the anhedonia phenotype depended on sex: the three-way (genotype × sex × concentrations) interaction achieved statistical significance [*F*_(2,30)_ = 3.79, *p* < 0.05, *η*_*p*_^2^ = 0.20]. While female KI^+/−^ mice primarily exhibited anhedonia at 1% sucrose concentration, their male counterparts exhibited the same effect at 0.25% sucrose concentration (see Supplementary Figure S1). Separate analysis of daily total fluid consumption did not reveal any difference between genotypes over the four test days. Their respective average daily fluid intake was WT = 5.65 ± 0.20 g; KI^+/−^ = 5.13 ± 0.17 g; and KI^+/+^ = 5.50 ± 0.21 g.

#### 3.1.2 FST

As depicted in Figure 3C, a progressive increase in immobility time was evident both within and between days. As expected, the 2-day FST paradigm allowed us to capture the expected pronounced increase in immobility in the early phase (1–4 min) on day 2 compared with that on day 1. It was during this early phase on day 2 that the separation between the mutants (KI^+/−^ and KI^+/+^) and WT was most notable. In contrast, this separation was visibly less obvious on day 1. To capture the complete evolution of immobility over the 2-day test, a 3 × 2 × 10 (genotype × days × 1-min bins) ANOVA of percentage time immobile was performed, which yielded a significant effect of genotype with a large effect size [*F*_(2,33)_ = 4.81, *p* < 0.05, *η*_p_^2^ = 0.23]. The earlier increase in immobility on day 2 also led to the significant effect of days [*F*_(1,33)_ = 9.97, *p* < 0.005, *η*_p_^2^ = 0.23] and days × bins interaction [*F*_(9,297)_ = 32.99, *p* < 0.001, *η*_p_^2^ = 0.50]. However, no interaction involving genotype achieved statistical significance.

*Post hoc* Fisher’s LSD tests confirmed that both heterozygous (KI^+/−^; *p* = 0.006) and homozygous mutants (KI^+/+^; *p* = 0.025) exhibited higher levels of immobility than WT mice, with no significant difference between KI^+/+^ and KI^+/−^ mice. To minimise confounds from initial motor agitation (especially on day 1), a supplementary analysis was conducted, restricted to the last 6 minutes (see right panel of Figure 3D). This did not alter the outcome. The genotype effect, which remained significant [*F*_(2,33)_ = 3.50, *p* < 0.05, *η*_p_^2^ = 0.18], and *post hoc* comparison again showed that KI^+/−^ and KI^+/+^ mice exhibited greater immobility than WT mice [*p* = 0.02 and 0.04, respectively] over this observation period (5–10 min). Similar to the overall analysis, all interaction terms involving genotype were non-significant. Thus, the observed phenotypic difference did not stem solely from the early phase (min 1–4) of the FST. As expected, exclusion of bins 1 to 4 from the analysis curtailed the ability to capture the faster emergence of immobility from day 1 to day 2 in the early phase: neither the main effect of days nor the days × bins interaction was significant.

#### 3.1.3 LH

There was no evidence of behavioural differences between genotypes based on the 2 × 2 × 2 × 5 (genotype × pre-exposure × days × blocks of 10 trials) ANOVA of response latency or percentage escape failures in conditioned active avoidance learning. As expected, mice in the yoked (inescapable) condition performed poorly, exhibiting longer shuttle latency [*F*_(1,66)_ = 10.20, *p* < 0.005, *η*_*p*_^2^ = 0.13] and more escape failures [*F*_(1,66)_ = 5.33, *p* < 0.05, *η*_*p*_^2^ = 0.08] compared with mice in the escapable condition. This deficit was comparable across genotypes. Neither the main effect of genotype nor its interaction reached statistical significance, suggesting that the mutation did not significantly modify the impact of prior loss of control over aversive events (learnt helplessness) on motivational behaviour.

Throughout avoidance training, learning was evidenced by a decrease in latency to shuttle across days [*F*_(1,66)_ = 143.71, *p* < 0.001, *η*_*p*_^2^ = 0.69] and across blocks of 10 trials [*F*_(4,264)_ = 42.27, *p* < 0.001, *η*_*p*_^2^ = 0.39] (data not shown). As expected within-day learning was more pronounced on Day 1 than on Day 2 [days × blocks interaction: *F*_(4,264)_ = 5.95, *p* < 0.001, *η*_*p*_^2^ = 0.08] in latency to shuttle. Initial analyses further confirmed that these statistical outcomes were consistent across sex and between cohorts A and B, despite their divergent behavioural histories.

### 3.2 Anxiolysis-like phenotype associated with *LRRK2* ^R1441G^ KI

#### 3.2.1 EPM

**FIGURE 4:**
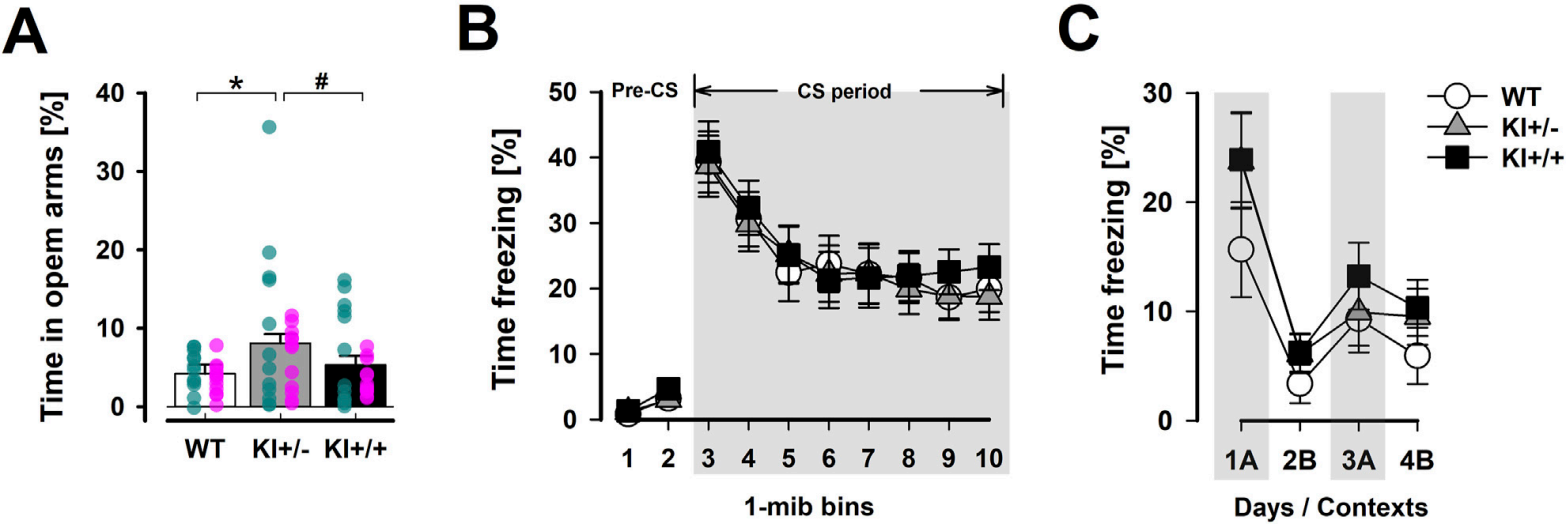
Selected phenotypes in anxiety-related behaviour. **(A)** The distribution of time spent in the open arms of the EPM relative to time in all arms (in percent) provides a measure of anxiety-like behaviour. A significant difference was detected using Fisher’s LSD test between KI^+/−^ and WT mice (*), while the difference between KI^+/−^ and KI^+/+^ did not achieve significance (# at *p* = 0.09). The EPM test included mice in both cohort A and B. n = 24 (12♀+ 12♂) per genotype. Overlaid scatter points represent data from individual mice (green dots = ♂; pink dots = ♀). **(B)** The expression of conditioned fear to the tone-CS, assessed 48 h after tone–shock pairings, was indexed by percentage time freezing in the tone test. Tone-freezing was continuously tracked for 8 min after the initial 2-min pre-CS period. The data presented are averaged across three consecutive test days (separate plots for each day are presented in Supplementary Figure S2B). **(C)** The expression of foreground contextual fear conditioning was assessed by returning the animals to the shocked context “A” on the first and third days (1A and 3A) and the neutral context “B” on the second and fourth days (2B and 4B) after conditioning. Higher levels of freezing in context “A” relative context “B” supported the expression of context-specific conditioned fear response. For **(B,C)**, n = 12 (6♀+ 6♂) per genotype. All data are presented as the mean ± SE.

A phenotype resembling anxiolytic drugs was observed in the heterozygous KI^+/−^ mice in the EPM test, while such an effect was absent in the homozygous KI^+/+^ mice (Figure 4A). This impression was supported by the significant main effect of genotype in the one-way ANOVA of percentage time spent in open arms [*F*_(2,69)_ = 3.04, *p* = 0.05, *η*_*p*_^2^ = 0.81]. An initial two-way (genotype × sex) ANOVA showed that this phenotype did not depend on sex. *Post hoc* comparisons confirmed that only KI^+/−^ mice spent significantly more time in the open arms than WT mice (*p* < 0.05). The KI^+/+^ genotype did not significantly differ from either of the other two genotypes, suggesting it may be associated with an intermediate phenotype. The difference between the heterozygous and homozygous KI groups did not achieve statistical significance (*p* = 0.09). There was no evidence of confounding changes in locomotor activity between genotypes. Total distance moved on the entire maze floor was comparable between genotypes: WT = 14.85 ± 0.78 m; KI^+/−^ = 13.60 ± 0.84 cm; KI^+/+^ = 13.86 ± 0.73 m. The difference between KI^+/−^ and KI^+/+^ mice reached significance only under a one-tailed criterion of *p* = 0.09, indicating that additional statistical power may be required to reliably differentiate them in this behavioural test. Finally, there was no evidence of confounding changes in locomotor activity between genotypes. Total distance moved across the entire maze floor was comparable: WT = 14.85 ± 0.78 m; KI^+^/^−^ = 13.60 ± 0.84 m; KI^+^/^+^ = 13.86 ± 0.73 m.

#### 3.2.2 Pavlovian fear conditioning

Evaluation of Pavlovian fear conditioning did not reveal an anxiolysis-like phenotype, as was suggested by the ethological anxiety test using the EPM. In Cohort A, conditioned freezing to both the discrete tone CS (Figure 4B) and the background context (Supplementary Figure S2A) in the cue-based fear conditioning paradigm was comparable between mutant and WT mice. Similarly, acquired freezing in the foreground context fear conditioning paradigm (Cohort B; Figure 4C) did not reliably distinguish mutant genotypes from WT.

For the tone-CS test, a 3 × 3 × 8 (genotype × day × 1-min bins) ANOVA of CS freezing across the three test days (days 3–5) did not reveal any significant genotype effect [all *F’*s < 1] (Figure 4B). Extinction learning was evidenced by the decrease in freezing across days [*F*_(2,66)_ = 37.47, *p* < 0.001, *η*_*p*_^2^ = 0.53] (Supplementary Figure S2B), within days [bins effect: *F*_(7,231)_ = 22.79, *p* < 0.001, *ηp* ^*2*^ = 0.41], and their interaction [*F*_(14,462)_ = 5.82, *p* < 0.001, *η*_*p*_^2^ = 0.15]. Analysis of freezing when these animals were re-exposed to the background context (Day 2), where tone–shock pairings had taken place, also revealed highly comparable levels of freezing over the 8-min exposure [mean % time freezing: WT = 13.30 ± 4.70, KI^+/−^ = 11.46 ± 3.14, and KI^+/+^ = 12.89 ± 4.17] (see Supplementary Figure S2A).

In the absence of a reliable discrete CS contingent with US presentation, foreground contextual conditioning also did not reveal any significant divergence between genotypes. All three genotypes exhibited conditioned freezing specific to the shock-paired context (A) relative to the neutral context (B). A rebound of freezing was clearly observed upon re-exposure to Context A for the second time (i.e., the 2B→3A transition in Figure 4C). The genotype × contexts ANOVA yielded the expected main effect of contexts [*F*_(1,33)_ = 22.33, *p* < 0.001, *η*_*p*_^2^ = 0.40], but provided no evidence for a genotype effect. When the analysis was restricted to Context A freezing, the genotype effect remained far from significance [*F* < 1]. The mean % time freezing across the two Context A exposures (‘1A’ and ‘3A’ in Figure 4C) was 12.48 ± 3.56 for WT, 16.84 ± 3.78 for KI^+/−^, and 18.57 ± 3.89 for KI^+/+^.

All findings described above were independent of sex, as confirmed by initial analyses that included the sex factor (data not shown).

### 3.3 Absence of effects in hippocampus-dependent memory tests

#### 3.3.1 Y-maze test of spatial familiarity judgement

**FIGURE 5:**
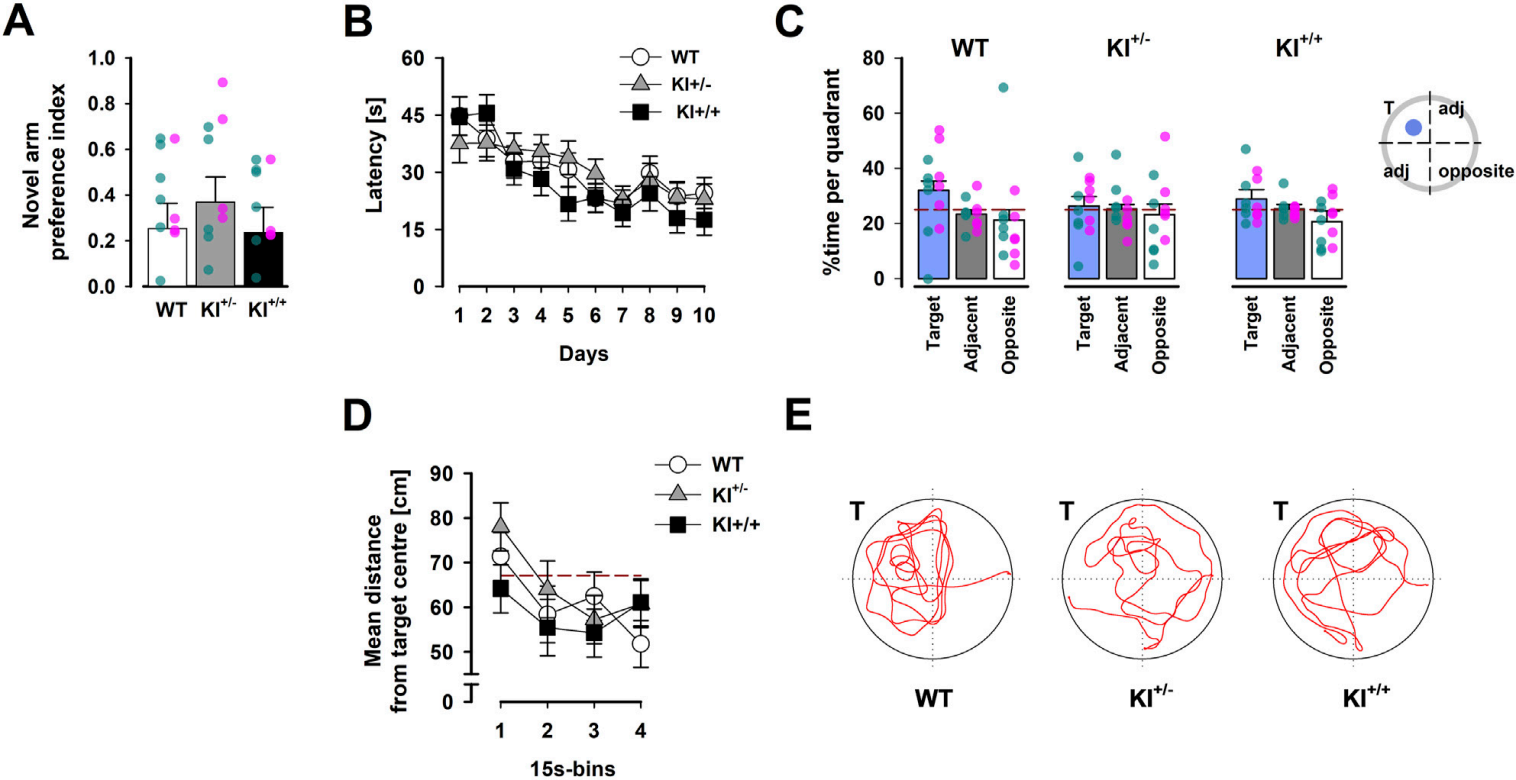
Hippocampus-dependent memory performance. **(A)** Preferential exploration of the novel arm in the Y-maze was indexed by a preference ratio, with values above 0 indicating a preference for the novel arm, and indifference at 0. Overlaid scatter points represent data from individual mice (green dots = ♂; pink dots = ♀). **(B)** Learning over the acquisition phase was evidenced by the reduction in latency across 10 days, which was comparably observed in mice of all three genotypes. **(C)** The proportion of time spent inside the target quadrant (in which the learnt platform was located during acquisition) is compared with that in the opposite quadrant and the two adjacent quadrants (expressed as their average) recorded in the probe test conducted 24 h after acquisition phase. The reference (dashed) line refers to chance performance at 25%. **(D)** The average distance between the mouse and the target centre showed a progressive decline over the course of the probe test. The reference (dashed) line represents chance performance (67.12 cm from target centre) estimated by Monte Carlo simulation. All data are presented as the mean ± SE. n = 12 (6♀+ 6♂) per genotype. **(E)** Swim paths recorded during the probe test correspond to mice of the three genotypes. “T” indicates the target quadrant.

No significant difference between genotypes was observed during the test phase when the preference for the novel arm, based on relative time spent [N/(N + F)], was subjected to a one-way ANOVA [*F* < 1; see Figure 5A], which confirmed the presence of an overall preference for the novel arms [*F*_(1,33)_ = 21.33, *p* < 0.001, *η*_*p*_^2^ = 0.39]. Separate analyses confirmed the lack of a genotype difference in time spent (in seconds) in the familiar arm in the sample phase [*F* < 1; WT = 114.62 ± 15.96, KI^+/−^ = 107.05 ± 9.86, KI^+/+^ = 105.56 ± 8.20]. Preliminary analyses revealed no influence of sex on Y-maze performance.

#### 3.3.2 Spatial reference memory test in the Morris water maze

No difference between genotypes was observed across all phases of the test. All mice swam and readily climbed onto the platform to escape from the water during pre-training with a visible platform.

Over the next 10 days, when the platform was hidden in a fixed location, acquisition of reference memory was evident by the reduction in escape latency (see Figure 5B) and distance travelled (see Supplementary Figure S3B) over days. This led to a significant days effect in separate 3 × 10 (genotype × days) ANOVA of latency [*F*_(9,297)_ = 10.56, *p* < 0.001, *η*_*p*_^2^ = 0.24] and path length [*F*_(9,297)_ = 9.98, *p* < 0.001, *η*_*p*_^2^ = 0.23]. Neither the main effect of genotype nor its interactions with days approached statistical significance [all *F*’s < 1]. There were no confounding swim speed differences between genotypes. The swim speed analysis showed that all effects related to genotypes were not significant.

In the probe test, comparison of the time spent searching in the target, adjacent, and opposite quadrants gave the visual impression that KI^+^/^−^ mice exhibited the weakest spatial search bias towards the target quadrant (Figure 5C). However, this impression was not supported by the presence of a significant interaction in the 3 × 3 (genotype × quadrant positions) ANOVA. Only the quadrants effect approached significance [*F*_(2,66)_ = 2.99, *p* = 0.06, *η*_*p*_^2^ = 0.08]. A clearly significant quadrants effect was detected in the parallel ANOVA of relative path length [*F*_(2,66)_ = 4.03, *p* < 0.05, *η*_*p*_^2^ = 0.11] (see Supplementary Figure S3C).

To assess the temporal dynamics of spatial search in the probe test, we also computed the average distance to the centre of the learnt target location across successive 15-s bins. A clear reduction in this metric over time was evident [*F*_(3,99)_ = 8.33, *p* < 0.02, *η*_*p*_^2^ = 0.2], without any indication of a gross difference between genotypes (Figure 5D). Neither the main effect of genotype nor the genotype × bins interaction approached statistical significance.

Finally, no significant difference in swim speed was detected between genotypes [WT = 24.14 ± 1.15 cm/s; KI^+/−^ = 22.83 ± 1.87 cm/s; and KI^+/+^ = 25.64 ± 0.74 cm/s], which was similar to the acquisition phase. Initial analyses of all variables above confirmed the absence of any significant sex-dependent effects of genotype (data not shown).

### 3.4 Normal expression of social behaviour in *LRRK2* ^R1441G^ KI mice

**FIGURE 6:**
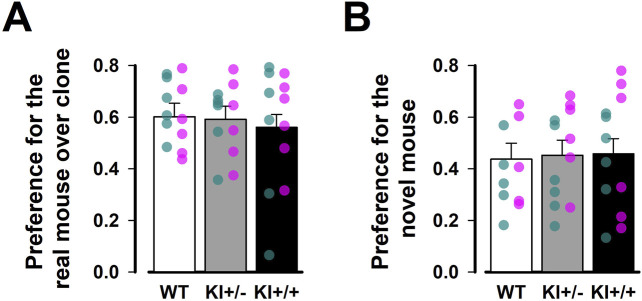
Three-chamber test of social behaviour. Social preference was assessed in **(A)** Social interaction phase and **(B)** Social novelty phase. Preferential exploration was indexed by a preference ratio, with values above 0.5 indicating preference for the chamber containing a live mouse over the one with the toy mouse during the social interaction phase, or for the chamber containing the novel mouse over the one with the familiar mouse during the social novelty phase. Values below 0.5 indicate the reverse preference in each phase, while a value of 0.5 indicates indifference. Data are presented as the mean ± SE. n = 12 (6♀+ 6♂) per genotype. Overlaid scatter points represent data from individual mice (green dots = ♂; pink dots = ♀).

There was no evidence of genotypic divergence in social behaviour, as assessed by the three-chamber sociability test (Figure 6). During the acclimatisation phase, mice of all three genotypes readily explored all chambers of the apparatus. Subsequently, a strong preference for the chamber containing a conspecific over the one containing a toy clone was similarly observed across genotypes (Figure 6A). In the subsequent social novelty phase, we did not observe an overall preference for the chamber housing the novel mouse (with a preference index <0.5), suggesting that our protocol was not sufficiently sensitive to detect social novelty. Against this background, no difference between genotypes was detected in this phase (Figure 6B). The genotype effect in the separate one-way ANOVAs of the respective preference indices derived from the two test phases was far from statistical significance [all *F’*s < 1].

### 3.5 Sensorimotor gating was unaffected by *LRRK2* ^R1441G^ KI

The whole-body startle reaction obtained from the pulse-alone trials (across pulse stimulus intensities: 100, 110, or 120 dB) was comparable across genotypes (data not shown). The 3 × 3 (genotype × pulse intensity) ANOVA of the startle reactivity yielded only a significant pulse intensity effect [*F*_(2,66)_ = 60.58, *p* < 0.001, *η*_*p*_^2^ = 0.65]; neither the genotype effect nor its interaction approached significance [both *F’*s < 1].

**FIGURE 7:**
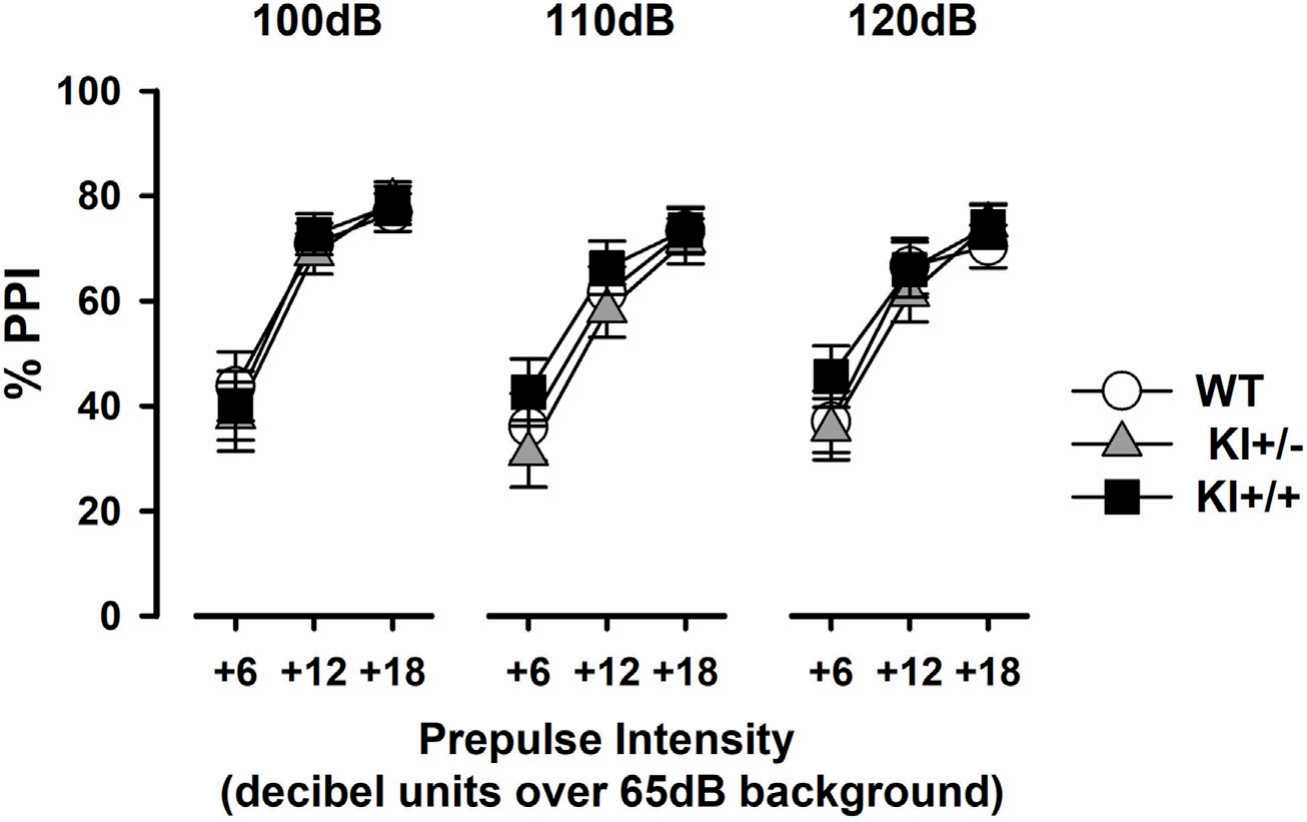
Prepulse inhibition of the acoustic startle reflex. Prepulse inhibition was indexed by %PPI and expressed as a function of increasing prepulse intensity (6, 12, or 18 dB units above constant background noise at 65 dB). Data are shown separately for each pulse intensity (100 dB, 110 dB, and 120 dB) and presented as the mean ± SE. n = 12 (6♀+ 6♂) per genotype.

PPI was evident in all mice as prepulses of increasing intensity led to stronger inhibition of the pulse-elicited startle response (expressed as a percentage reduction, %PPI), regardless of pulse stimulus intensity (Figure 7). A 3 × 3 × 3 (genotype × pulse intensity × prepulse intensity) ANOVA of %PPI confirmed a monotonic increase in %PPI with increasing prepulse intensity [*F*_(2,66)_ = 290.45, *p* < 0.001, *η*_*p*_^2^ = 0.90] but yielded no evidence of a genotype effect or its interactions. In line with previous research (Csomor et al., 2008; Yee et al., 2005), the pulse intensity effect also reached statistical significance [*F*_(2,66)_ = 6.24, *p* < 0.005, *η*_*p*_^2^ = 0.16], with %PPI being stronger at lower 100 dB pulse intensity than at 110 dB (*p* < 0.005) and 120 dB (*p* < 0.05).

### 3.6 Limited impact on open-field activity, muscle strength, and motor coordination

#### 3.6.1 Open-field locomotor activity

**FIGURE 8:**
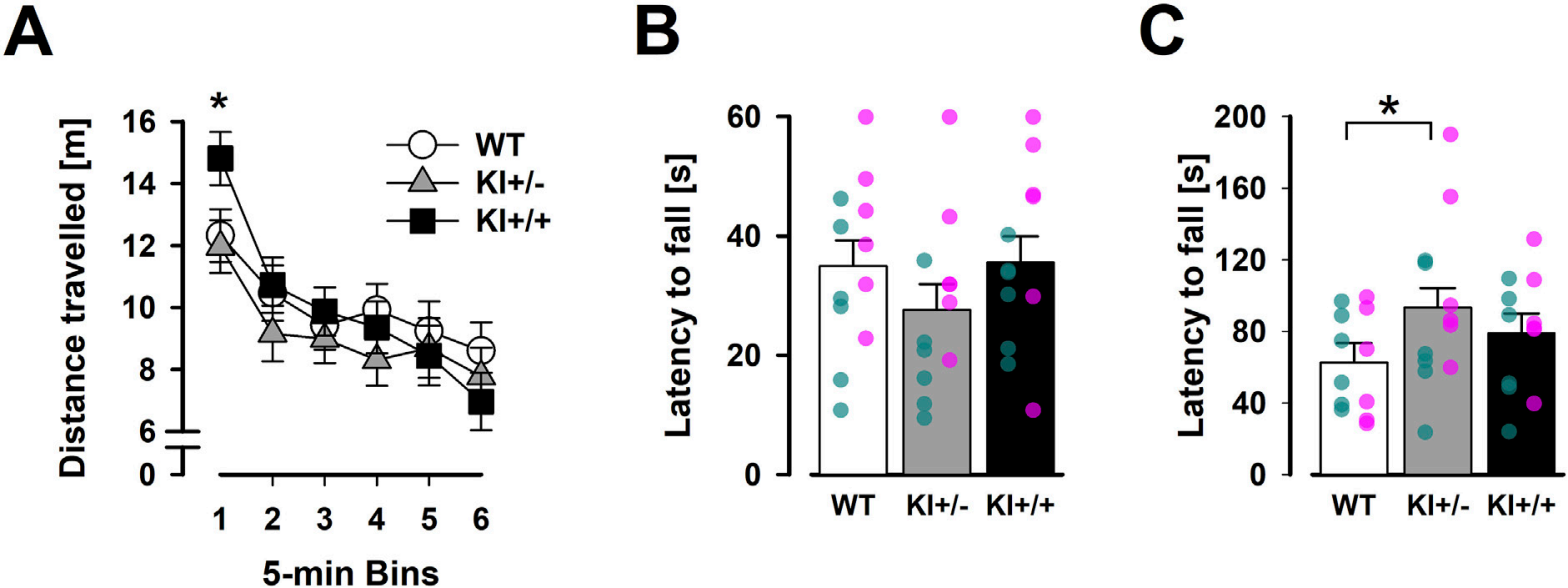
Results of motor behaviours (OF, inverted grid, and accelerating rotarod). Spontaneous locomotor activity is plotted against successive 5-min time bins in **(A)**. The first 5-min bin labelled with “*” indicates a significant difference between KI^+/+^ and other two genotypes based on Fisher’s LSD (vs. KI^+/+^, *p* < 0.001; vs. WT, *p* < 0.001). The average latency to fall in three trials of the inverted grid test **(B)** and accelerating rotarod **(C)** are shown. “*” indicates the significant *ad hoc* Fisher’s LSD comparison (*p* < 0.05). Data are presented as the mean ± SE. n = 12 (6♀+ 6♂) per genotype. Overlaid scatter points represent data from individual mice (green dots = ♂; pink dots = ♀).

In the open field, KI^+/+^ mice exhibited an initial elevation of locomotor activity during the first 5 min. Afterwards, mice of all three genotypes remained highly comparable until the end of the open-field test, including in the expression of locomotor habituation (Figure 8A). Although the main effect of genotype was not significant [*F* < 1], the 3 × 6 (genotype × 5-min bins) ANOVA of distance moved yielded a significant interaction [*F*_(10,165)_ = 1.9, *p* < 0.05, *η*_*p*_^2^ = 0.10], confirming the time-dependent activity phenotype in KI^+/+^ mice. The overall presence of locomotor habituation also led to a highly significant bins effect [*F*_(5,165)_ = 27.88, *p* < 0.001, *η*_*p*_^2^ = 0.46].

The absence of a genotype × bins interaction in a supplementary ANOVA restricted to the last 25 min (bins 2–6) of the open-field test suggested that the KI^+/+^ phenotype was transient rather than indicative of a more persistent impact on locomotor habituation. This KI^+/+^ phenotype also appeared to be context-dependent as no comparable hyperlocomotion phenotype was detected in the 5-min EPM (see Section 3.2). The more anxiety-provoking context of the EPM might have masked this phenotype. Conversely, analysis of thigmotaxis (average minimal distance from the arena walls) in the open field did not reveal any genotype differences [*F* < 1], providing no evidence for the anxiolysis-like phenotype previously observed in KI^+/−^ mice in the EPM.

#### 3.6.2 Grip strength and motor coordination

Separate 3 × 3 (genotype × trials) ANOVAs of latency to fall in the inverted grid and accelerating rotarod tests yielded neither a genotype effect nor a genotype × trials interaction. A motor learning effect was detected only in the accelerating rotarod, as indicated by a significant trials effect [*F*_(2,66)_ = 6.14, *p* < 0.005, *η*_*p*_^2^ = 0.24] (data not shown), with no evidence of differential expression between genotypes. Nonetheless, visual inspection suggested that KI^+/−^ mice fell earlier in the inverted grid test (Figure 8B) but remained on the rotarod for longer (Figure 8C) than WT mice. The latter impression, which was inconsistent with motor deficiency, was statistically supported by an *ad hoc* Fisher LSD comparison [*p* < 0.05]. Overall, our results did not suggest the presence of gross motor impairments in our KI mice.

## 4 Discussion

This study provides the most comprehensive assays of non-motor behaviours in the *LRRK2* ^R1441G^ KI mouse line to date. The inclusion of both heterozygous and homozygous mutants was instrumental in revealing the presence of depression-related traits: anhedonia in the SPT and avolition in the FST. These phenotypes are relevant to affective symptoms in PD, particularly in carriers of the *LRRK2* ^R1441G^ mutation. Given that LRRK2 mutations in PD patients are typically heterozygous, our results may better approximate the situation in this population (Gorostidi et al., 2009; Vinagre-Aragón et al., 2021).

The affective phenotypes were largely independent of sex. Notably, they were consistently observed in the heterozygous KI^+/−^ genotype. The anxiolysis-like phenotype (in the EPM test) was also uniquely present in KI^+/−^ mice although its relevance to PD-related affective dysfunction is questionable. In contrast, the homozygous KI^+/+^ genotype was only associated with one affective phenotype reported in the FST, which is clearly not stronger (and arguably slightly weaker) than that observed in KI^+/−^ mice.

The apparent preponderance of affective and emotional effects of the heterozygous KI genotype, coupled with the partial penetrance of the homozygous KI genotype, is inconsistent with a simple gene dosage effect or haploinsufficiency (Whiffin et al., 2020). Instead, this nonlinear genotype–phenotype relationship may indicate the possible involvement of a dominant-negative mechanism, whereby the heterozygous state uniquely harbours both mutant and wild-type LRRK2 proteins, enabling aberrant intermolecular interactions that are essentially absent in the homozygous state (Ohta et al., 2013). This would be critical since LRRK2 normally functions as a dimer (Civiero et al., 2017). The dominant-negative effect could be mediated by the formation of mutant/WT LRRK2 heterodimers that interfere with normal kinase function and downstream signalling cascades (Ohta et al., 2013). This, in combination with compensatory changes, threshold effects, and context-dependent penetrance, may collectively drive the behavioural phenotypic profile observed in KI^+/−^ mice. Such complex interaction is worth considering in future attempts to build an integrative account of the diverse phenotypic outcomes across genetic mouse models (see Table 1). Dissecting the mechanistic links between specific LRRK2 mutations and early non-motor symptoms in Parkinson’s disease is key to clarifying how distinct genetic models converge or diverge in their pathogenic trajectories.

Next, we examine the behavioural manifestations of the R1441G KI mouse line in detail, beginning with a focused analysis of the affective phenotypes that are central to the non-motor symptoms of Parkinson’s disease.

### 4.1 Affective phenotypes

The prominent depression-related phenotypes observed in heterozygous *LRRK2* ^R1441G^ knock-in (KI^+/−^) mice align closely with the presence of anhedonia and volitional deficits in up to 70% of patients with PD (Angelopoulou et al., 2023; Kalinderi et al., 2024; Weintraub et al., 2022). Although neither the KI^+/−^ nor the KI^+/+^ genotype significantly affected the sensitivity to develop learnt helplessness in the avoidance task, we noted that the impact of inescapable shock pre-exposure appeared visually stronger in KI than in WT mice, especially in terms of escape failure (Figure 5C). Hence, we cannot rule out the possibility that a significant genotype effect might emerge if a more severe regimen of shock pre-exposure had been employed. The novel depression-related phenotypes demonstrated in this study, however, contrast with anti-depressant-like behaviours reported in the R1441C knock-in line (Giesert et al., 2017; Table 1). Nonetheless, our data support the notion that the R1441 domain is capable of modulating depression-related behaviour in the context of PD. The demonstrated link of the R1441G mutation with depression in this study also aligns with the numerical difference in the incidence of depression among R1441G carriers compared to non-carriers (14.8% vs. 0%) reported by Bergareche et al. (2016) even though this epidemiological study was underpowered. On the other hand, our findings in the FST and SPT aligned well with the G2019S transgenic model, including their independence of sex (Lim et al., 2018; Table 1), although Kim et al. (2022) later showed that the depression-like phenotypes were specific to the female mice only when evaluated at the adolescent age (7–9 weeks old). The convergence of depression-like phenotypes in our R1441G KI^+/−^ mice with those in the G2019S transgenic model reinforces the critical role of LRRK2 mutations in PD non-motor psychopathology, warranting deeper investigation into their shared mechanisms (e.g., dysregulated dopaminergic and/or serotonergic signalling and neuroinflammation) and potential sex-dependent developmental trajectories.

In the study, the only hint of sex-dependency was the anhedonia phenotype observed in the SPT. It was necessary to vary the sucrose concentration in order to reveal the mutation’s effect on sensitivity to reward magnitude and discrimination between reward levels (Bolaños et al., 2008; Markov, 2022). Male KI^+/−^ mice exhibited a steeper decrease in sucrose preference when sucrose concentration was reduced from 1% to 0.25%, whereas female KI^+/−^ mice already showed a weaker preference at 1% concentration. This sex difference may stem from heightened sensitivity to changes in reward intensity in the male mutants but reduced perception of reward value in the female mutants. Hence, the possibility that the R1441G mutation interacts with sex-specific neural mechanisms to produce distinct anhedonia-like behaviours cannot be entirely excluded. It is, therefore, worth noting that similar sex-dependent effects have been reported in other LRRK2 mutation models, albeit with inconsistent outcomes (Giesert et al., 2017; Kim et al., 2022).

While the anhedonia phenotype revealed by the SPT was solely observed in the KI^+/−^ mice, the volitional deficit observed in the FST was notable in both KI^+/−^ and KI^+/+^ mice. Both KI^+/−^ and KI^+/+^ mice exhibited significantly higher immobility than WT throughout the 2-day FST protocol, which was visually the strongest during the early onset of Day 2 (*cf.* Porsolt et al., 1977). The FST results clearly do not conform to a simple gene dosage effect or haploinsufficiency as KI^+/−^ and KI^+/+^ mice were statistically indistinguishable in their expression of floating. However, our interpretation of these co-occurring phenotypes is potentially undermined by the use of the same cohort for both behavioural assays. We cannot rule out the possibility that the SPT phenotype was driven by an anomalous response to FST-induced stress, representing a behavioural extension of the FST phenotype. For such a transfer effect to account for the observed results, it would need to selectively affect the KI^+/−^ mice, given the absence of a consistent SPT phenotype in KI+/+ mice. We, therefore, consider this explanation unlikely and inconsistent with a parsimonious account of the data. To conclusively rule it out, future studies should counterbalance test order or employ separate cohorts for SPT and FST.

In comparison with our *LRRK2* ^R1441G^ KI line, the *LRRK2* ^G2019S^ KI model has highlighted more complex interactions with environmental stress. Despite not exhibiting overt baseline behavioural differences from WT, homozygous G2019S KI male mice apparently displayed bi-direction modulation of social avoidance behaviour following social defeat stress. Both resilience and sensitisation (relative to WT response) have been demonstrated (see Table 1) depending on whether prior social defeat was experienced for 10 days or 1 day, respectively (Matikainen-Ankney et al., 2018; Guevara et al., 2020). This nuance was, however, not apparent in terms of pleasure-related (hedonic) response. Both reports showed data pointing to a stronger sucrose preference in G2019S KI^+/+^ following social defeat even though the effect was reportedly significant only after the 1-day social defeat procedure (Guevara et al., 2020). Unfortunately, we may not take into account the mutated gene dosage effect when comparing behavioural profiles between G2019S and R1441G knock-in models because these two studies of the G2019S KI model evaluated only the homozygous G2019S genotype.

### 4.2 Anxiolytic-like phenotypes

Contrary to the observed prevalence of anxiety in PD, the phenotype identified in the EPM test suggested the presence of an anti-anxiety effect, which was demonstrated free from concerns over confounding changes in locomotor activity (Walf and Frye, 2007; Weiss et al., 1998). However, it is not entirely unexpected. Ho et al. (2023) had previously reported a substantial reduction in marble-burying behaviour, resembling the response to anxiolytic drugs (Broekkamp et al., 1986), in the R1441G KI mouse line. Furthermore, a non-significant trend toward reduced anxiety was also visible (but not significant) in the EPM for R1441G transgenic mice (Bichler et al., 2013, see their Figure 3). Similarly, Ho et al. (2023) also reported an anti-anxiety phenotype in homozygous R1441G KI^+/+^ mice, although we did not observe a parallel effect in these homozygous mutant mice (but only in the heterozygous, KI^+/−^, mutants). Hence, to some degree, anxiety reduction (as opposed to anxiety heightening) appears to be a robust effect of the R1441G mutation. The anxiolysis phenotype was apparently specific to the innate response to ethological stimulus signalling environmental danger because the expression of conditioned fear was unaffected. Learning to attribute fearful qualities to previously neutral discrete or contextual cues *via* Pavlovian association was unaffected by the R1441G mutation.

Taken together, R1441G mouse models could enjoy face validity for PD-related depression but not PD-related anxiety (Abou Kassm et al., 2021). This apparent decoupling between threat sensitivity and motivational persistence may represent a psychological profile marked by diminished avoidance behaviour but impaired goal-directed effort. Phenomenologically, this dissociation could reflect a shift in behavioural strategy, where reduced avoidance in the EPM signifies not only decreased anxiety but also heightened exploratory drive or altered risk appraisal (Carobrez and Bertoglio, 2005). Conversely, increased immobility in the FST could indicate a lower threshold for passive coping, suggesting active disengagement rather than classical behavioural despair (Commons et al., 2017; West, 1990). This pattern resembles apathy syndromes in some PD patients, characterised by reduced initiative and engagement independent of overt dysphoria or anxiety (Levy and Dubois, 2006; Pagonabarraga et al., 2015).

To explore whether the depressive-like and anxiolytic-like behaviours co-varied at the individual level, we conducted an *ad hoc* correlational analysis within the KI^+/−^ group. It revealed that avolition and anxiolysis phenotypes did not positively correlate. Instead, KI^+/−^ mice with higher immobility time in the FST tended to spend less time in the open arms of the EPM (i.e., displaying more anxiety-like behaviour) [*r* = −0.35, *p* = 0.26, *df* = 10]. This non-significant relationship aligns with the observed comorbidity of anxiety and depression in both the PD population (see Weintraub et al., 2022) and rodent models (Gencturk and Unal, 2024), but further testing with a larger sample space would be necessary. Taken together, the co-expression of depressive-like and anxiolytic-like phenotypes in KI^+/−^ mice may reflect distinct neuropsychological dysregulations and may not be surprising in light of certain theoretical formulations (Willner, 1997). Our data further raise the possibility that the R1441G mutation may confer resilience to anxiety, as hinted by an earlier small sample survey of PD patients, in which anxiety symptoms were detected in 4.5% carriers of R1441G compared to 15% among non-carriers (Estanga et al., 2014). We encourage sufficiently powered replications to clarify the precise contribution of the R1441G mutation to the prevalence of anxiety symptoms in PD.

Although no study has yet quantified or characterised serotonergic signalling specifically in the context of the R1441G mutation, alterations in 5-HT1A receptor expression have been reported in limbic structures in association with the G2019S mutation, potentially influencing anxiety and mood regulation (Lim et al., 2018). It would be interesting to clarify if similar serotonergic changes could contribute to the reduced anxiety-like behaviour observed in our KI^+/−^ mice. Another speculative link may involve the disruption of macroautophagy and mitophagy and subsequent impairment in mitochondrial function and increased oxidative stress (Yakhine-Diop et al., 2022). Metabolically demanding brain regions, such as the prefrontal cortex and dorsal striatum, could be more prone to the negative impact of compromised energy homeostasis, resulting in blunted motivation and behavioural perseverance, which manifests as increased immobility in the FST. Although highly speculative, serotonergic modulation and mitochondrial vulnerability may act through distinct neural pathways to produce the atypical combination of anxiolytic and depressive traits in our *LRRK2* ^R1441G^ KI model.

Finally, a broader evaluation of the anxiolytic-like phenotype using paradigms sensitive to anxiety (e.g., the partial reinforcement extinction effect and differential reinforcement of low rates of responding; Gray, 1982; Gray and McNaughton, 2000) is certainly warranted to better characterise its context-dependent penetrance.

### 4.3 Lack of gross motor phenotypes

Our *LRRK2* ^R1441G^ KI mice lack gross motor function impairment, but a significant genotype × bins interaction was observed in the open-field test. The homozygous KI^+/+^ mutants appeared to show a steeper habituation slope, primarily driven by higher initial locomotor activity. This transient hyperactivity may be linked to R1441G-induced dopaminergic dysregulation reported previously (Liu et al., 2014), which is distinct from the more robust affective phenotypes in KI^+/−^ mice. Notably, no similar hyperactivity was evident in the EPM, suggesting its context-dependency. Conversely, the increased rotarod latency in KI^+/−^ mice may indicate enhanced motor coordination. This impression, however, may not be the exception as enhanced motor performance has been reported before in other mutant mouse models (Herzig et al., 2012; Skiteva et al., 2022; Yue et al., 2015). Nonetheless, neither of these phenotypes resembles PD-like motor deficits, reinforcing our suggestion that the other behavioural changes observed in our mutant model are not secondary to motor impairments. The segregation of these motor phenotypes between homozygous and heterozygous KI mice and their differences in robustness and persistence suggest that they stem from distinct underlying mechanisms.

### 4.4 Absence of hippocampal-dependent cognitive and other phenotypes

In contrast to the depression and anxiety phenotypes observed, our study found no evidence of cognitive deficits in R1441G KI mice, assessed through tasks like the Morris water maze, Y-maze, and Pavlovian associative conditioning. Similarly, prior studies reported no or minimal cognitive deficits in transgenic R1441G, knock-in R1441C, and knock-in G2019S mice, tested between 2 and 12 months of age (see Table 1). In contrast, transgenic models overexpressing either *LRRK2* ^G2019S^ or *LRRK2* ^R1441C^ exhibited a deficit in T-maze spontaneous alternation at 18–21 months old but not 3–6 months old (Sloan et al., 2016). This suggested that age could be a crucial determinant. The possibility that the emergence of memory deficits could be detected at middle age was supported by Adeosun et al. (2017), who reported the presence of radial-arm water maze and Y-maze impairments in 9–10-month-old G2019S transgenic mice. Hence, our inability to detect hippocampus-dependent memory deficit in our mutants could be due to their relatively young age at the time of testing (between 4.5- and 7-months old during the Y-maze and Morris water maze). It is necessary to qualify our assertion of domain-specific phenotypes in our R1441G KI must be restricted to early adulthood.

Intact performance in the Morris water maze and Y-maze in our mutants also does not preclude the presence of cognitive impairments in other non-hippocampus-dependent domains, particularly tasks that rely on fronto-striatal circuits, which are more prominently affected in the early stages of PD (Dirnberger and Jahanshahi, 2013; Williams-Gray et al., 2007). Fronto-striatal deficits, such as impaired executive function, working memory, and attentional set-shifting, are well-documented in early PD and may better reflect the cognitive profile associated with LRRK2 mutations (Robbins and Cools, 2014). Future studies should incorporate tasks, e.g., attentional set-shifting paradigms, to evaluate fronto-striatal-dependent cognitive functions in the LRRK2^R1441G^ KI model, thereby providing a more comprehensive cognitive assessment. Relevant domains affected in the G2019S KI model include visuospatial attention (evaluated by the 5-choice serial reaction time task, 5-CSRTT) and instrumental conditioning. Deficits in both have been demonstrated in 2-3-month-old G2019S KI mice (Hussein et al., 2022).

Beyond the two hippocampus-dependent memory tests, we also included the PPI test of sensorimotor gating due to its sensitivity to striatal dopaminergic dysfunction and hippocampal neurochemical imbalance (Swerdlow et al., 1992; Bast and Feldon, 2003). PPI is commonly used to assess pre-attentive filtering deficits associated with diverse neuropsychiatric conditions in animals and humans (Swerdlow et al., 2016; Santos-Carrasco and De la Casa, 2023; Hazlett et al., 2008; Yee, 1999). Although the majority of studies are related to schizophrenia, deficient PPI expression has also been reported in patients with PD and dementia with Lewy bodies (Gabrielli et al., 2002; Perriol et al., 2005), which is further linked to poorer attention, slower psychomotor speed, and reduced dopamine reuptake in the caudate (Zoetmulder et al., 2014). Notably, psychotic symptoms, including hallucinations and delusions, are increasingly recognised throughout the course of PD (Pagonabarraga et al., 2024; Zahodne and Fernandez, 2008; Chang and Fox, 2016). There is also a suggestion that psychotic symptoms in the prodromal phase may predict a higher risk of transition to PD (Pachi et al., 2021). However, evidence linking LRRK2 mutations to psychotic symptoms in PD, particularly in the prodromal phase, remains very limited. The absence of PPI deficits in our R1441G KI mice aligns with an earlier report in a transgenic R1441G rat model (Shaikh et al., 2015). The null effect is consistent with the absence of striatal hyperdopaminergia in this model (Liu et al., 2014), suggesting that the R1441G mutation is unlikely to contribute to sensory flooding linked to psychotic symptoms in schizophrenia (Swerdlow et al., 2008). This conclusion aligns with clinical studies indicating that the R1441G and G2019S mutations are not associated with a significant increase in psychotic symptoms in PD (Gaig et al., 2014). Thus, the R1441G mutation may not be a promising target to mitigate psychotic symptoms in PD.

Given that emotions and affect play a substantial role in social behaviour (Moore and Isen, 1990), the absence of social behavioural deficits in R1441G KI mice is noteworthy. It reinforces the mutation’s apparent specificity for depression-like and anxiety-related phenotypes, without broader impact on social approach or novelty preference. In contrast, the *LRRK2* ^G2019S^ KI model has shown altered social avoidance following both acute and chronic social stress (Matikainen-Ankney et al., 2018; Guevara et al., 2020). The lack of social phenotypes in R1441G KI mice may suggest that the dopaminergic alterations associated with this mutation (Ho et al., 2023) are more relevant to affective domains than to social functioning. Longitudinal assessments could help determine whether effects emerge over time, while additional tasks (such as those probing emotional recognition and empathic responsiveness) may be required to detect subtler social deficits, which are known to be impaired in PD (Alonso-Recio et al., 2021; Argaud et al., 2018).

Overall, to better characterise potential cognitive decline associated with the R1441G mutation, behavioural assessments should be extended into old age. This would help reveal any additional age-dependent phenotypic shifts in our KI model.

### 4.5 Differential phenotypic penetrance between heterozygous and homozygous LRRK2^R1441G^ mutation

**FIGURE 9:**
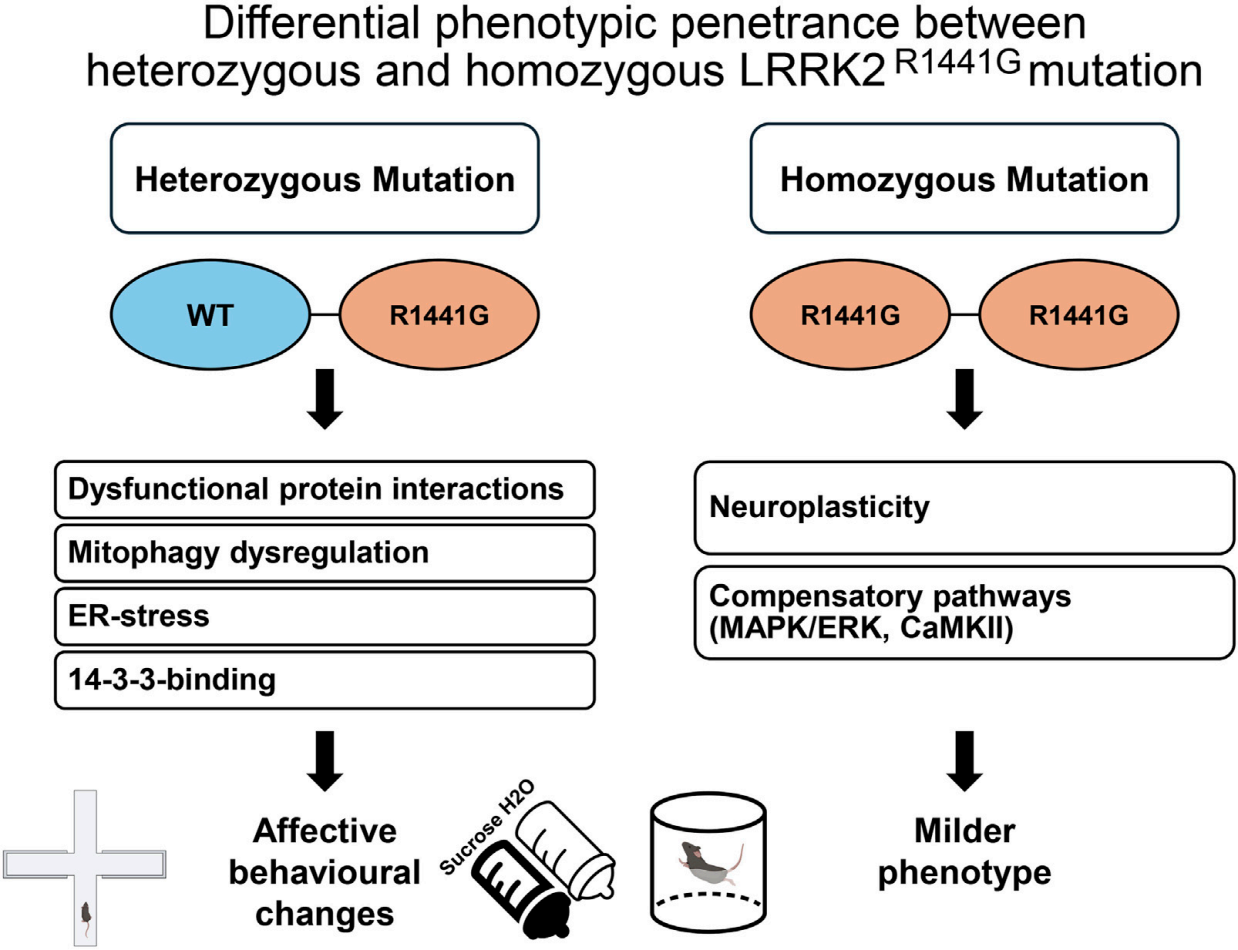
Schematic representation of the differential phenotypic penetrance in KI^+/−^ *versus* KI^+/+^ LRRK2^R1441G^ KI mice. In KI^+/−^ mice, co-expression of WT and mutant (R1441G) proteins promotes dysfunctional heterodimer formation, leading to aberrant protein interactions that may amplify mutation-induced molecular disturbances (i.e., mitophagy dysregulation, endoplasmic reticulum [ER] stress, and impaired 14-3-3 binding). These disturbances could drive the observed affective behavioural changes, including anxiolysis-like behaviour in the EPM, anhedonia in the SPT, and avolition in the FST. In contrast, KI^+/+^ mice exclusively form R1441G homodimers, which may activate compensatory mechanisms such as neuroplasticity and upregulation of alternative signalling pathways (e.g., MAPK/ERK or CaMKII), yielding a milder phenotype. This model illustrates the hypothesised dominant-negative effect in heterozygotes and adaptive responses in homozygotes, underlying the differential phenotypes observed in the present study.

The predominance of behavioural phenotypes in KI^+/−^ mice over their KI^+/+^ littermates challenges the simple gene dosage effect, which predicts stronger or at least comparable effects in homozygous KI^+/+^ mice with two mutated alleles. Our KI+/− mice displayed anxiolysis-like behaviour in the EPM and anhedonia in the SPT, whereas KI^+/+^ mice exhibited a slightly weaker depression-like phenotype in the FST but not anhedonia in the SPT. This non-linear genotype–phenotype relationship suggests that the co-expression of WT and mutant LRRK2^R1441G^ proteins in KI^+/−^ mice drives dysfunctional protein interactions (Figure 9). One speculation is the formation of LRRK2 heterodimers between WT and mutant proteins (R1441G/WT dimers) in the heterozygous KI^+/−^ mice. Given that dimerisation is a critical determinant of LRRK2 kinase activity (Leandrou et al., 2019), the co-expression of *LRRK2* ^WT^ and *LRRK2* ^R1441G^ is expected to modify LRRK2 function as a dimer. The severity of behavioural phenotypes could be determined by the balance between homodimers (WT/WT and R1441G/R1441G) and R1441G/WT heterodimers. Ohta et al. (2013) demonstrated that I2020T/WT LRRK2 heterodimers impair cellular functions, such as kinase activity and anti-apoptotic protection, and hypothesised a dominant-negative effect resulting from the interaction between mutant and wild-type subunits in heterodimers. The mutation of the R1441 site in the Ras of complex (ROC) domain primarily affects GTPase activity, and its mutations are expected to alter dimer stability or interactions (Deng et al., 2008). Yakhine-Diop et al. (2022) further demonstrated that the R1441G mutation disrupts mitophagy and induces endoplasmic reticulum (ER) stress—cellular disturbances that may be amplified by the presence of R1441G/WT heterodimers in KI^+/−^ mice. Furthermore, aberrant protein–protein interactions and post-translational modifications can contribute to dysregulated LRRK2 function by disrupting membrane association and complex assembly (Berwick et al., 2019; Chang et al., 2022). Proper LRRK2 function critically depends on phosphorylation at S1444. Its impairment disrupts 14-3-3 protein binding and compromises kinase regulation (Muda et al., 2014), thereby destabilising LRRK2’s interactions with regulatory partners. These altered molecular interactions (potentially compounded by disrupted dimerisation dynamics) can impair key functional domains, including kinase activity, GTPase regulation, and downstream signalling cascades (Civiero et al., 2012; Ohta et al., 2013). In KI^+/−^ mice, the formation of R1441G/WT heterodimers may play a pivotal role in precipitating the early onset of affective behavioural abnormalities observed in this study.

In addition, the milder phenotype in KI^+/+^ mice may imply compensatory mechanisms that effectively attenuate the impact of the R1441G mutation in the homozygous state. For instance, alternative signalling pathways regulating neuronal plasticity, such as MAPK/ERK or CaMKII (Boon et al., 2014), may be upregulated, potentially activating redundant systems like other leucine-rich repeat kinases or calcium-dependent kinases to partially restore affective function (Chang et al., 2022; Giaime et al., 2017; Hinkle et al., 2012). R1441G/R1441G homodimers may also adopt a conformation that partially preserves LRRK2 function, thus limiting the toxic interaction with regulatory proteins, mitophagy dysregulation or ER stress compared to the destabilising effects of R1441G/WT heterodimers (Berwick et al., 2019). The constitutive expression of *LRRK2* ^1441G^ could also trigger neuroplastic developmental changes in the brain (e.g., prefrontal cortex or amygdala) through enhanced synaptic plasticity, altered neurotransmitter release (e.g., dopamine or serotonin), or increased inhibitory control (Bichler et al., 2013). Moreover, cellular homeostasis mechanisms, such as upregulated autophagy pathways or ER stress response proteins (e.g., BiP), may counteract R1441G-induced toxicity in KI^+/+^ mice, preserving neuronal function and reducing behavioural deficits (Ho et al., 2020; Yuan et al., 2011). These multiple compensatory mechanisms likely act in concert, with homodimer-specific properties, pathway upregulation, and circuit-level adaptations collectively mitigating the phenotype in KI^+/+^ mice, whereas the dominant-negative interference of R1441G/WT heterodimers in KI^+/−^ mice may preclude such adaptations.

To strengthen our interpretation, we considered whether alternative mechanisms might better account for the observed genotype–phenotype disparity. First, a toxic gain-of-function model, where the R1441G mutant protein independently drives cellular dysfunction through aberrant kinase activity or substrate hyperphosphorylation, would predict a more severe phenotype in KI^+/+^ mice with two mutant alleles. The milder KI^+/+^ phenotype, coupled with evidence of compensatory mechanisms, argues against this model and supports the dominant-negative hypothesis, where R1441G/WT heterodimer interference exacerbates the heterozygous phenotype. Second, a nonlinear allele-dose effect, whereby the heterozygous state represents a critically precise intermediate threshold that disrupts affective signalling without triggering compensatory changes, is theoretically possible but less parsimonious. Third, haploinsufficiency, where a single wild-type allele in KI^+/−^ mice is insufficient to maintain normal function, does not fit the differential phenotypic penetrance observed. It would rather predict a more severe phenotype in KI^+/+^ mice. The *LRRK2* ^R1441G^ protein is functional with altered properties suggestive of active interference rather than a loss of function (Civiero et al., 2012; Ohta et al., 2013). The lack of affective phenotypes in LRRK2 knockout mice (Volta et al., 2015) further challenges explanations based on haploinsufficiency, which also does not take into account the dimer-dependent nature of LRRK2 function outlined above. In contrast, the dominant-negative framework emphasises heterodimer interaction as critical. However, it has not escaped our notice that this emphasis appears inconsistent with the absence of overt behavioural effects in transgenic models overexpressing *LRRK2* ^R1441G^ (Bichler et al., 2013; Shaikh et al., 2015; see Table 1). One plausible explanation is that the overwhelming abundance of R1441G/R1441G homodimers in these transgenic models may trigger compensatory responses, potentially promoted by intrinsic properties specific to the homodimers themselves.

Since only the downstream effects of homozygous R1441G KI have been examined to date (Chang et al., 2022; Liu et al., 2021; Ho et al., 2017), further characterisation of R1441G heterodimer function is necessary to clarify its role in potential dominant-negative phenotypes. A deeper understanding of LRRK2 dimerisation dynamics may also present a therapeutic opportunity to restore the balance between heterodimers and homodimers, potentially modulating disease progression in Parkinson’s patients with LRRK2-linked pathogenesis (Deyaert et al., 2017). Co-immunoprecipitation would be needed to confirm R1441G/WT heterodimer formation and assess its impact on kinase activity in KI^+/−^ mice. RNA sequencing and electrophysiological recordings would be instrumental in identifying upregulated compensatory pathways at the cellular level and adaptations at the circuit level.

## 5 Conclusion

Taken together, our findings show that heterozygous *LRRK2* ^R1441G^ knock-in mice exhibit robust affective non-motor phenotypes, which are attenuated in homozygous and overexpression models. This specific genotype–phenotype mapping highlights the heterozygous KI model’s utility for recapitulating early non-motor symptoms and reflecting the genetics of most human carriers. The higher penetrance of these affective behaviour changes in heterozygotes leads us to speculate on a dominant-negative mechanism, driven by LRRK2 heterodimer dynamics, as a promising axis for understanding how the R1441G mutation drives affective dysfunction. On this basis, disrupted upstream and downstream signalling pathways, compensatory synaptic-plasticity adaptations, and intrinsic homodimer properties likely converge to shape these outcomes. These insights position the model as a valuable tool for dissecting non-motor pathology. Future studies can probe heterodimer-specific mechanisms to guide mutation-informed precision therapies for LRRK2-linked PD. At the clinical level, longitudinal studies in R1441G and G2019S carriers (charting symptom progression and tracking biomarkers) will be essential to translate these preclinical insights into patient care.

## Data Availability

The raw data supporting the conclusions of this article will be made available by the authors, without undue reservation.
