# Supplementary material for: Affective phenotypes in heterozygous LRRK2 R1441G knock-in mice
Source: Front Genet. 2025 Aug 29;16:1629897. doi: 10.3389/fgene.2025.1629897 (PMC12426193; doi:10.3389/fgene.2025.1629897)
Supplement: Supplementary file 1 [file DataSheet1.pdf]

Supplementary Material

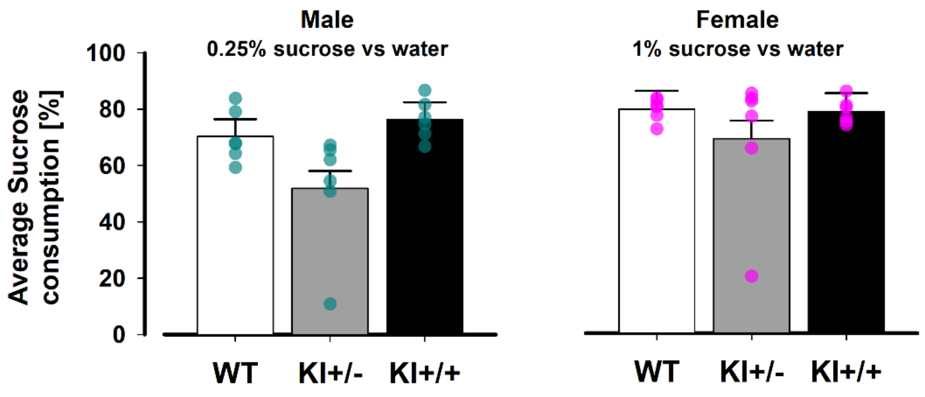

Supplementary Figure S1. The expression of anhedonia-like behaviour in male and female mice was primarily seen in the test conducted with the sucrose solution at 1% and 0.25%, respectively, against plain water. Overlaid scatter points represent data from individual mice (green dots =♂; pink dots =♀).

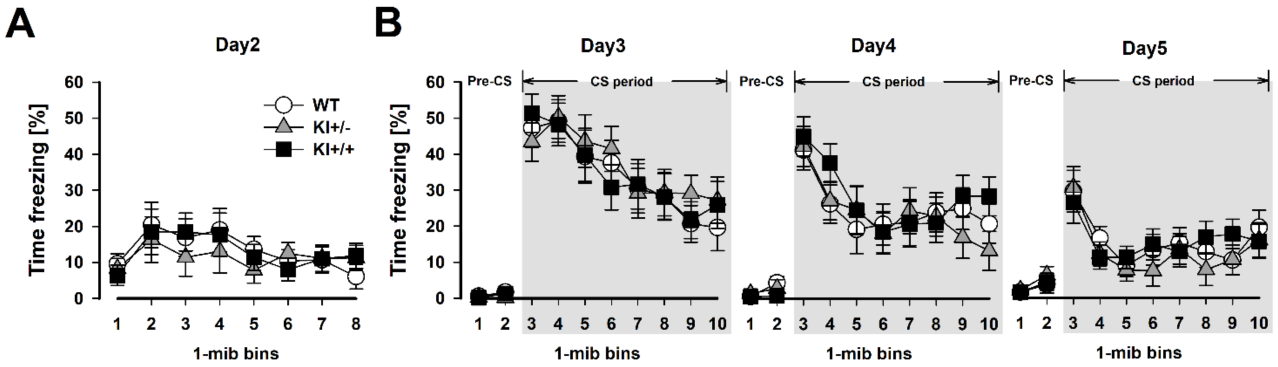

Supplementary Figure S2. Freezing time data recorded during (A) 8-min context test on Day 2 & (B) tone-CS test on three consecutive days (Day 3, Day 4, Day 5).

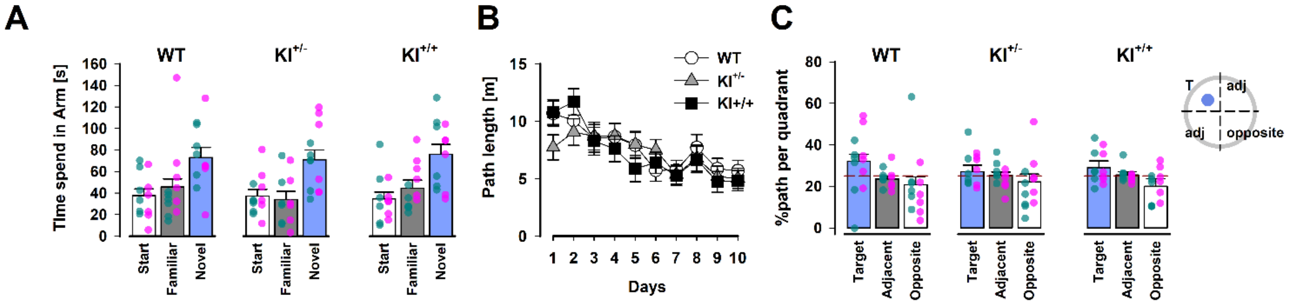

Supplementary Figure S3. **(A)** Averaged time spent in Start, Familiar and Novel arms during the Y-Maze test session are presented separately for three genotypes. The preference index, calculated as (N−F)/(N+F) using time spent in the Familiar and Novel arms, is presented in **Section 3.3, Fig 5A**. **(B)** Performance of spatial reference memory test in the water maze, indexed by path length over the 10 days of acquisition. **(C)** The proportion of path length across three quadrant positions: the target, the two adjacent (average), and the opposite quadrants. The statistical outcome resembled the analyses of time presented in **Section 3.3.** Overlaid scatter points represent data from individual mice (green dots =♂; pink dots =♀).

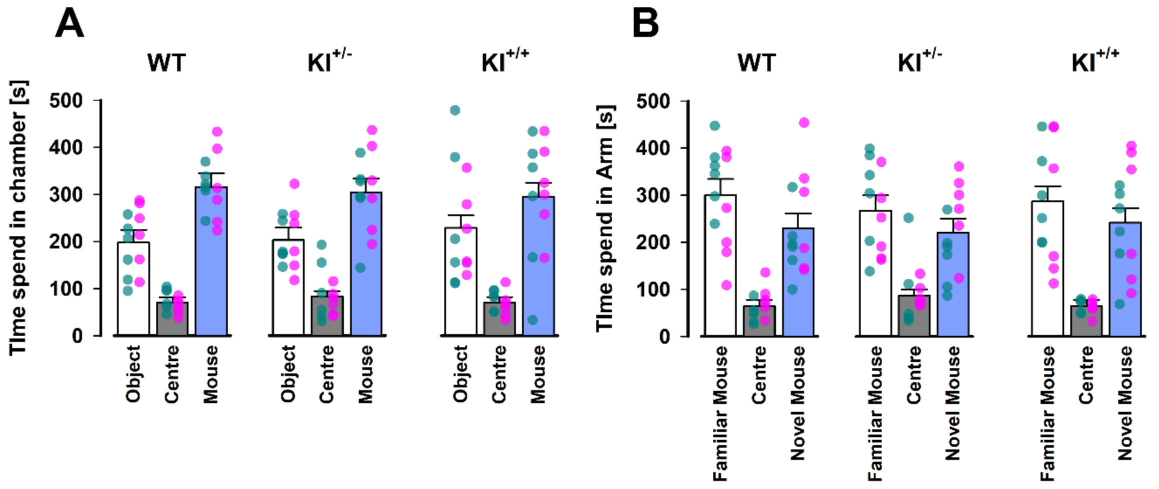

Supplementary Figure S4. Averaged time spent in each chamber during the three-chamber social behaviour test, presented separately for the **(A)** social interaction phase and **(B)** social novelty phase. Preference ratios, calculated as (mouse)/(mouse + toy) for social interaction and (unfamiliar)/(unfamiliar + familiar mouse) for social novelty, were used to index relative preferences and are analysed in **Section 3.4 (see Fig 6)**. Overlaid scatter points represent data from individual mice (green dots =♂; pink dots =♀).
